# OSMAC Method to Assess Impact of Culture Parameters on Metabolomic Diversity and Biological Activity of Marine-Derived Actinobacteria

**DOI:** 10.3390/md22010023

**Published:** 2023-12-28

**Authors:** Alexandre Le Loarer, Laurent Dufossé, Jérôme Bignon, Michel Frédérich, Allison Ledoux, Mireille Fouillaud, Anne Gauvin-Bialecki

**Affiliations:** 1 Laboratory of Chemistry and Biotechnology of Natural Products, Faculty of Sciences and Technology, University of La Réunion, 15 Avenue René Cassin, CS 92003, CEDEX 09, 97744 Saint-Denis, France; alexandre.le-loarer@univ-reunion.fr (A.L.L.); laurent.dufosse@univ-reunion.fr (L.D.); mireille.fouillaud@univ-reunion.fr (M.F.); 2 Institute of Chemistry of Natural Substances (ICSN), CNRS UPR 2301, Université Paris-Saclay, 1, av. de la Terrasse, 91198 Gif-sur-Yvette, France; jerome.bignon@cnrs.fr; 3 Pharmacognosy Laboratory, Department of Pharmacy, Centre Interfacultaire de Recherche sur le Médicament (CIRM), University of Liège, Campus du Sart-Tilman, Quartier Hôpital, Avenue Hippocrate, 15, B36, 4000 Liege, Belgium; m.frederich@uliege.be (M.F.); allison.ledoux@uliege.be (A.L.)

**Keywords:** OSMAC method, *Salinispora*, *Micromonospora*, marine-derived actinobacteria, molecular network, specialized metabolites, cytotoxic activity, antiplasmodial activity

## Abstract

Actinobacteria are known for their production of bioactive specialized metabolites, but they are still under-exploited. This study uses the “One Strain Many Compounds” (OSMAC) method to explore the potential of three preselected marine-derived actinobacteria: *Salinispora arenicola* (SH-78) and two *Micromonospora* sp. strains (SH-82 and SH-57). Various parameters, including the duration of the culture and the nature of the growth medium, were modified to assess their impact on the production of specialized metabolites. This approach involved a characterization based on chemical analysis completed with the construction of molecular networks and biological testing to evaluate cytotoxic and antiplasmodial activities. The results indicated that the influence of culture parameters depended on the studied species and also varied in relation with the microbial metabolites targeted. However, common favorable parameters could be observed for all strains such as an increase in the duration of the culture or the use of the A1 medium. For *Micromonospora* sp. SH-82, the solid A1 medium culture over 21 days favored a greater chemical diversity. A rise in the antiplasmodial activity was observed with this culture duration, with a IC_50_ twice as low as for the 14-day culture. *Micromonospora* sp. SH-57 produced more diverse natural products in liquid culture, with approximately 54% of nodes from the molecular network specifically linked to the type of culture support. Enhanced biological activities were also observed with specific sets of parameters. Finally, for *Salinispora arenicola* SH-78, liquid culture allowed a greater diversity of metabolites, but intensity variations were specifically observed for some metabolites under other conditions. Notably, compounds related to staurosporine were more abundant in solid culture. Consequently, in the range of the chosen parameters, optimal conditions to enhance metabolic diversity and biological activities in these three marine-derived actinobacteria were identified, paving the way for future isolation works.

## 1. Introduction

For some decades now, researchers have intensified the exploration of biodiversity, particularly in marine environments, to discover new natural products with well-established bioactive properties [1,2]. Among these, microorganisms, and more specifically actinobacteria, are recognized for their production of bioactive specialized metabolites [3,4,5,6,7]. Despite the large number of bioactive microbial compounds currently isolated, advances in microbial genomics demonstrated a significant difference between the number of biosynthetic gene clusters (BGCs) dedicated to specialized metabolites and the number of molecules actually isolated by researchers [8,9]. These differences arise from “silent” BGCs under laboratory conditions, thereby limiting the full exploration of microbial chemical possibilities. Researchers tend to expand techniques to activate these silent BGCs [9,10], including the One Strain Many Compounds (OSMAC) method. It aims at modifying selected parameters, which can be physical (type of culture support or duration of growth) or chemical (composition of the growth medium or pH). Other methods include the use of chemical or biological elicitors [11,12,13]. These changes can induce the production of a higher number of microbial metabolites and lead to the discovery of new bioactive molecules [13,14].

In our study, three microbial strains, *Salinispora arenicola* SH-78, *Micromonospora* sp. SH-82, and *Micromonospora* sp. SH-57, isolated from the microbiota of the marine sponge *Scopalina hapalia* ML-263, were studied [15]. These strains belong to the actinobacteria phylum and, more specifically, to the *Micromonosporaceae* family. They are generally isolated from soils but can originate from different habitats such as the marine environment. They also form holobionts with eukaryotic organisms, such as sponges, seaweeds, mollusks, or corals [3,5,15]. This family is known to produce a variety of bioactive compounds [3,5,16], particularly the marine obligate genus *Salinispora*, with the production of Salinosporamide A, an anticancer compound currently under clinical investigation [17,18]. The present work develops the impact of selected culture parameters on the metabolic diversity of the microbial extracts and their associated biological activities. These three strains were preselected based on previous studies using a prioritization method or their anti-aging properties [15,19]. An OSMAC culture strategy was then set up to evaluate the impact of cultivation time, support of growth (liquid or solid), or culture medium composition on the production of microbial metabolites. The extracts obtained from the cultures were chemically analyzed, allowing for the creation of Ion Identity Molecular Networks (IIMNs) [20] and the annotation of specialized metabolites, thanks to bioinformatics tools [21,22,23]. A selection of microbial extracts, obtained from diverse culture conditions, was tested for their cytotoxic and antiplasmodial potential to assess the impact of the parameters on the biological activities and correlate them with chemical diversity. The results indicated that the influence of culture parameters was strain dependent and varied according to the microbial metabolites targeted. *Micromonospora* sp. SH-82 exhibited substantial production of specialized metabolites under several culture conditions. However, the use of the A1 medium and an extended culture duration led to the detection of a greater number of compounds. Liquid cultures of *Micromonospora* sp. SH-57 demonstrated higher potential for discovering new bioactive molecules, with increased biological activity, compared to solid media. Lastly, liquid cultures of *Salinispora arenicola* SH-78 highlighted a greater chemiodiversity. 

## 2. Results

The three microbial strains, *Micromonospora* sp. SH-82, *Micromonospora* sp. SH-57, and *Salinispora arenicola* SH-78, were cultivated under different culture parameters. Control cultures were conducted under similar conditions but without the inoculation of microorganisms. Standardized microbial extracts were analyzed by HPLC-CAD, allowing for a quantitative evaluation, and also by UHPLC-HRMS-MS, enabling the creation of Ion Identity Molecular Networks (IIMNs). The produced metabolites were identified using bioinformatics tools, and their key annotations were summarized as histograms for each culture condition. Finally, a selection of microbial extracts was tested for their antiplasmodial and cytotoxic activities. The extracts were considered remarkable regarding antiplasmodial activity if the inhibition percentage exceeded 50% at 10 µg/mL. Then, the concentration required to inhibit the growth of the parasite by 50% (IC_50_) was measured. The extracts were considered promising for cytotoxic activity if the viability was below 50% at a concentration of 1 µg/mL. 

### 2.1. Micromonospora sp. SH-82

#### 2.1.1. Influence of Culture Support and Time on *Micromonospora* sp. SH-82’s Productions 

*Micromonospora* sp. SH-82 was cultivated on A1 medium in two different supports (solid and liquid), over 7, 14, or 21 days. A total of six microbial crude extracts were obtained by ethyl acetate solvent extraction and analyzed to evaluate the chemical diversity and the production of bioactive metabolites.

##### Chemical Analysis

A minimum of 13 peaks were detected in the HPLC-CAD chromatograms of each extract, highlighting a substantial richness in metabolites (Table S1). The main observation is an increased number of visible (>5 pA) and major (>30 pA) peaks over time. Table S2 supports these statements with peak details. After 21 days, a higher number of major peaks was observed for the solid culture (12) compared to the liquid culture (7). Finally, five peaks were detected exclusively in the extracts from liquid culture and four peaks from solid culture.

**Figure 1:**
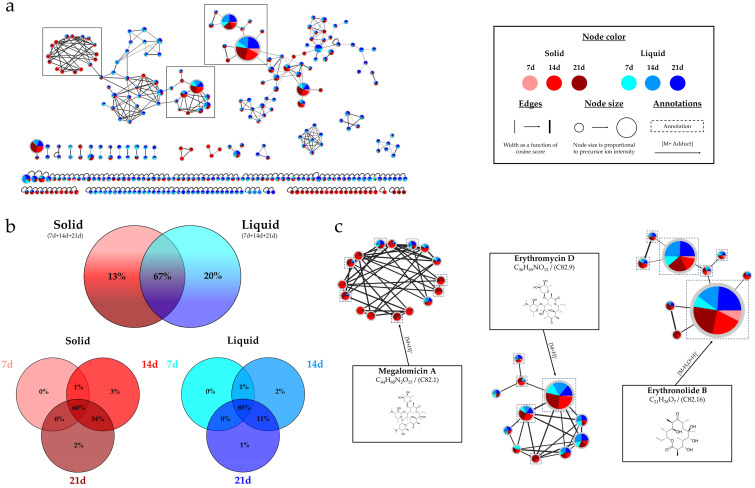
*Micromonospora* sp. SH-82: (**a**) Ion Identity Molecular Network (IIMN) from the extracts of A1 solid (red gradient) and liquid cultures (blue gradient) at 7, 14 and 21 days. (**b**) Percentages of nodes as a function of culture parameters (culture support and duration). (**c**) Zoom on 3 annotated clusters of interest.

Figure 1a illustrates the Ion Identity Molecular Network resulting from the high-resolution analysis of the extracts from *Micromonospora* sp. SH-82. The network includes a total of 315 nodes, 43% of which are grouped into 24 clusters containing more than 2 nodes. Two major clusters (>50 nodes) are observed in this network.

The molecular network exhibits an homogeneous distribution of nodes between the solid and liquid culture supports (Figure 1b). The top diagram shows that 67% are common to both solid and liquid supports, and 33% of unique nodes are almost evenly distributed between solid and liquid supports (13% and 20%, respectively). The other two diagrams provide a detailed view of the nodes from each support over time. It is observed that the number of nodes increased over time, particularly from the solid medium. Only 61% of the nodes are observed at 7 days, while 98% and 96% are detected at 14 and 21 days, respectively. Figure 1c is a zoomed-in view of three interesting clusters, with annotated nodes. The left one represents the megalomicins, with seven annotated and unique nodes specific to the solid medium that could not be identified. The middle cluster represents the erythromycins, and the right cluster lays out the erythronolides family, in which the annotated nodes were observed in all the studied extracts. 

**Figure 2:**
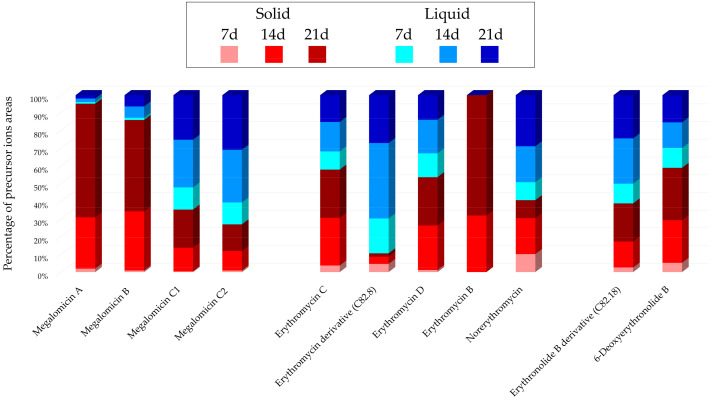
*Micromonospora* sp. SH-82: Main annotations in the IIMN designed from the extracts of the A1 solid and liquid cultures at 7, 14 and 21 days. The histograms present the cumulative proportions relative to the precursor ions areas intensities attributed to each culture condition.

Histograms in Figure 2 represent the main annotated nodes and their relative amounts in the different culture conditions. The amounts of the metabolites are reported as the sum of precursor ions areas.

Figure 2 demonstrates that the annotated precursor ions are generally visible in all the extracts. Except for erythromycin B, the six colors are indeed represented in each histogram. However, a difference is observed in intensities, based on the type of compound. For megalomicin A (C 82.1, *m*/*z* 877.5648 [M + H]^+^, C_44_H_80_N_2_O_15_) and megalomicin B (C 82.2, *m*/*z* 919.5754 [M + H]^+^, C_45_H_78_N_2_O_17_), the precursor ion intensity percentage is higher from solid cultures, accounting for over 85%, primarily originating from the 21-day growth (64% and 52%, respectively). However, for megalomicins C1 and C2, the indicated percentages are higher from liquid cultures (65% and 73%, respectively). For the other annotations, the distribution is scattered between solid and liquid supports, with the exception of erythromycin B (C 82.10, *m*/*z* 718.4717 [M + H]^+^, C_37_H_67_NO_12_), which is only detected from the extracts of 14- and 21-day solid cultures.

##### Bioassays

**Figure 3:**
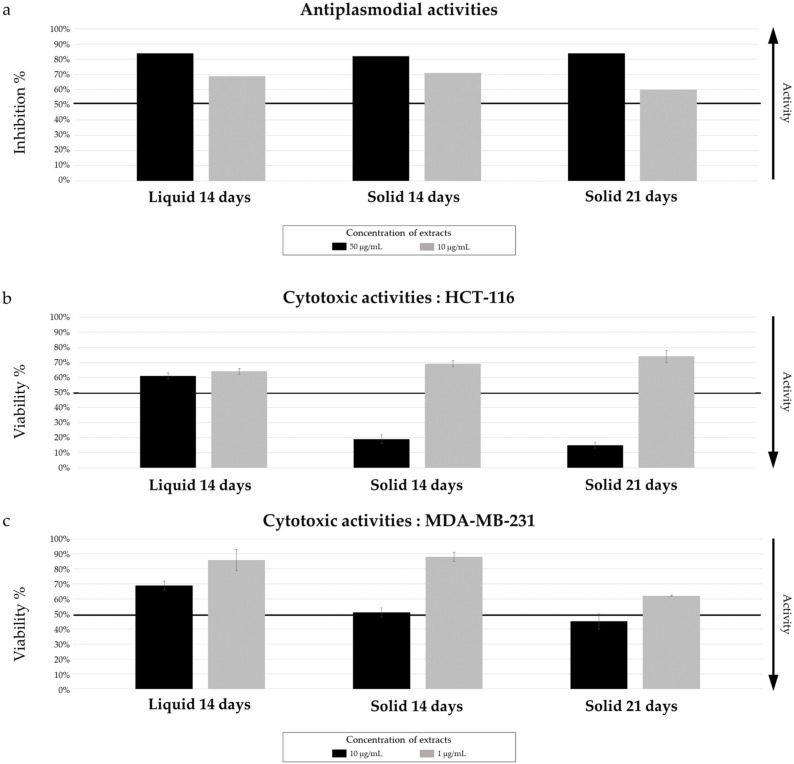
*Micromonospora* sp. SH-82: Biological activity of extracts from cultures on A1 solid and liquid media at 14 days and 21 days (solid media). (**a**) Antiplasmodial activity against *P. falciparum* strain 3D7, tested at 50 µg/mL and 10 µg/mL. (**b**) Cytotoxic activity against HCT-116 cell line and (**c**) MDA-MB-231 cell line, tested at 10 µg/mL and 1 µg/mL. The black lines indicate the threshold for considering the extract as promising: antiplasmodial activity >50% inhibition; cytotoxic activity <50% viability.

A selection of three extracts of *Micromonospora* sp. SH-82 was evaluated for their antiplasmodial (against *Plasmodium falciparum* 3D7 strain) and cytotoxic activities (against HCT-116 and MDA-MB-231 cell lines). The extracts tested allowed for a comparison between liquid and solid A1 medium cultures at 14 days and solid A1 medium cultures at 14 and 21 days (Figure 3).

The results for antiplasmodial activity, presented in Figure 3a, showed a similar activity between solid and liquid extracts at 14 days. The extract obtained from the solid culture demonstrated an IC_50_ of 11.31 ± 1.18 µg/mL versus 9.73 ± 4.07 µg/mL from liquid culture. Concerning the cytotoxic activity against both cell lines (Figure 3b,c), a lower cell viability is observed for the extract from the solid culture, at a concentration of 10 µg/mL. This observation does not hold true when the tested concentration is 1 µg/mL.

Concerning the impact of culture time (14 and 21 days on solid media), both extracts exhibited promising antiplasmodial activity (Figure 3a), with an inhibition percentage greater than 50% at 10 µg/mL. The extract from the 21-day culture had a lower IC_50_ (5.06 ± 0.95 µg/mL), compared to those from the 14-day culture (11.31 ± 1.18 µg/mL), making it more promising. Both extracts did not show very promising results towards cytotoxic activities. A lower cell viability against the MDA-MB-231 cell line was observed for the 21-day culture extract (Figure 3c). 

#### 2.1.2. Influence of Medium Composition and Culture Support on *Micromonospora* sp. SH-82’s Productions

*Micromonospora* sp. SH-82 was cultivated on A1 and MB (Marine Broth) media over 14 days, on two types of supports, solid and liquid. A total of four microbial crude extracts were analyzed. 

##### Chemical Analysis

From Table S1, a higher number of visible peaks was observed from the A1 medium at 14 days, regardless of the culture support, with an average of 20 peaks for extracts from the A1 medium and 13 peaks from the MB medium. However, the extract from the MB liquid culture showed a higher number of major compounds compared to the extract derived from the A1 liquid medium, with 10 and 5 major peaks, respectively. Four specific peaks were only visible from the liquid MB medium and solid A1 medium extracts (Table S2), and, typically, the peak heights were greater in the latter.

In the same way as Figure 1, an IIMN was created from the extracts studied (Figure S1a). The related network comprises a total of 253 nodes, 57% of which were grouped into clusters (>2 nodes). The blue and red colors, picturing the extracts from cultures on A1 medium, are predominant in the molecular network. The diagram on top (Figure S1b) confirms this observation, with 94% of the nodes originating from the A1 medium extract and 32% unique to this medium. Regarding the influence of the support, the diagram illustrating the MB medium showed a heterogeneous distribution of nodes between the liquid and solid supports. Further, 99% of the nodes came from the liquid MB medium, and 52% were unique to it. 

Similar to Figure 2, the same main annotations of this IIMN were displayed under the form of histograms (Figure S2). It pointed out that the majority of precursor ion areas primarily originated from the A1 culture extracts. However, a lower proportion was also observed from the extracts of MB cultures, indicating the presence of ions in all culture conditions. One node identified as erythromycin B (C 82.10, *m*/*z* 718.4717 [M + H]^+^, C_37_H_67_NO_12_) was specific to the extract derived from the solid A1 culture, and the node corresponding to norerythromycin (C 82.15, *m*/*z* 706.4521 [M + H]^+^, C_35_H_63_NO_13_) was predominant in the extract from the liquid MB culture.

##### Bioassays

The two ethyl acetate extracts from the 14-day solid cultures of *Micromonospora* sp. SH-82 on A1 and MB media were evaluated for their antiplasmodial and cytotoxic activities (Figure S3). For the antiplasmodial activity (Figure S3a), the MB culture medium extract exhibited higher inhibition percentages compared to A1 extracts and a lower IC_50_ measurement of 6.03 ± 2.10 µg/mL. As for the cytotoxic activity (Figure S3b,c), the extracts did not show very promising activities at a concentration of 1 µg/mL.

#### 2.1.3. Overview of the Annotations 

A synthesis of the annotations of the metabolites obtained from the extracts of *Micromonospora* sp. SH-82, cultivated under different conditions, is presented in Table 1. The raw formulas, the compound names or their InChiKey, the chemical class, and the similarity score are provided. Table S3 summarizes additional chemical details and precursor ion areas observed for each annotation in MZmine software 3.6.0, according to the culture conditions. 

A total of 21 nodes were successfully annotated, 7 of which led to similar identifications using two different bioinformatics tools. Among them, megalomicin C1 (C 82.4, *m*/*z* 961.5883 [M + H]^+^, C_48_H_84_N_2_O_17_), erythromycin B (C 82.10, *m*/*z* 718.4717 [M + H]^+^, C_37_H_67_NO_12_), and 6-deoxyerythronolide B (C 82.21, *m*/*z* 369.2626 [M-H_2_O+H]^+^, C_21_H_38_O_6_) were identified, thus allowing for the detection of the three main families of compounds present in the extracts. The relevant MS data are provided in the Supplementary Material (Figures S4–S6).

During these analyses, the microbial metabolites produced by *Micromonospora* sp. SH-82 formed a large number of doubly charged ions, not displayed in Figure 1a. They are shown in Figure S7, highlighting their high intensity.

The megalomicin ion intensity presented in Table S3 reinforces the observation that the support and the culture time influence the production of metabolites. Indeed, for 66% of the annotations, the maximum intensity area of precursors ions is observed for the extracts derived from 21-day cultures. The node annotated as 13-deethyl-13-methylerythromycin (C 82.8, *m*/*z* 690.4431 [M + H]^+^, C_36_H_65_NO_12_) exhibits higher precursor ion area intensity for the extracts obtained from A1 liquid cultures. Figure S8 displays a zoomed-in view of the cluster region encompassing this annotation, revealing the presence of numerous other nodes, predominantly colored in blue, which could indicate a favored occurrence on the solid support.

### 2.2. Micromonospora sp. SH-57

#### 2.2.1. Influence of Culture Support and Time on *Micromonospora* sp. SH-57’s Productions

In this part of the study, *Micromonospora* sp. SH-57 was cultivated in liquid or solid A1 medium over 7, 14, or 21 days. Further, six microbial crude extracts were obtained by ethyl acetate solvent extraction and analyzed to evaluate their chemical diversity and biological activity.

##### Chemical Analysis 

For this strain, the number of peaks detected by HPLC-CAD in all microbial extracts ranged from 4 to 10 (Table S1); this is lower compared to *Micromonospora* sp. SH-82. The first observation is an increased number of visible compounds over time, reaching a maximum at 21 days. For the extract derived from liquid culture, an increase of six peaks was observed between 14 and 21 days of culture. The second observation is an increase in four compounds in the 21-day liquid culture compared to solid culture at the same time (Table S4). In this liquid condition, three peaks are unique, and the intensity is greater than in the rest, indicating the possible presence of a higher quantity of microbial compounds.

**Figure 4:**
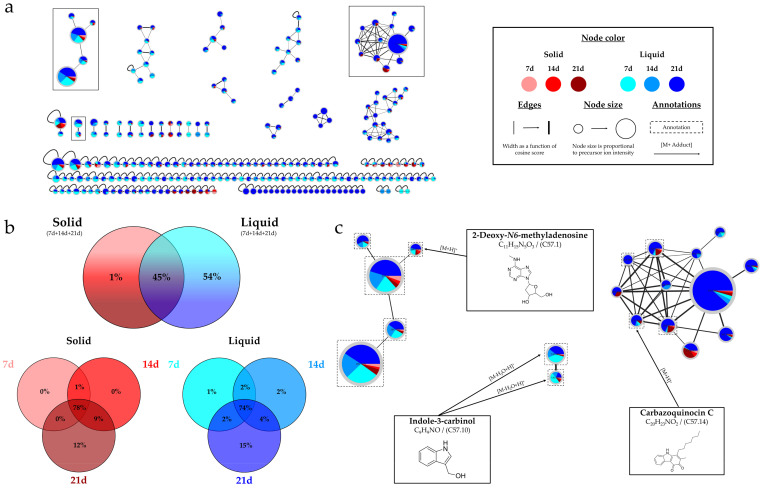
*Micromonospora* sp. SH-57: (**a**) Ion Identity Molecular Network (IIMN) from the extracts of A1 solid (red gradient) and liquid cultures (blue gradient) at 7, 14 and 21 days. (**b**) Percentages of nodes as a function of culture parameters (culture support and duration). (**c**) Zoom on 3 annotated clusters of interest.

The IIMN obtained from the cultures of *Micromonospora* sp. SH-57 highlights the influence of the culture support and time (Figure 4a). This network includes a total of 250 nodes, 40% of which are grouped into 24 clusters (>2 nodes).

The liquid cultures (blue in color) are predominant in Figure 4a. The top diagram in Figure 4b confirms this observation, with 99% of the nodes visible in the extracts from liquid cultures and 54% of them specific to it. The number of nodes also increases over time, with over 95% of nodes visible at 21 days, regardless of the support (Figure 4b bottom). The left cluster in Figure 4c, for which all nodes are annotated, corresponds to imidazopyrimide compounds. It shows a predominance of dark-blue color, indicating a greater presence of precursor ions from the extracts of 21-day liquid cultures. In the large right cluster, the same color predominates, with three nodes annotated as carbazoquinocin derivatives.

**Figure 5:**
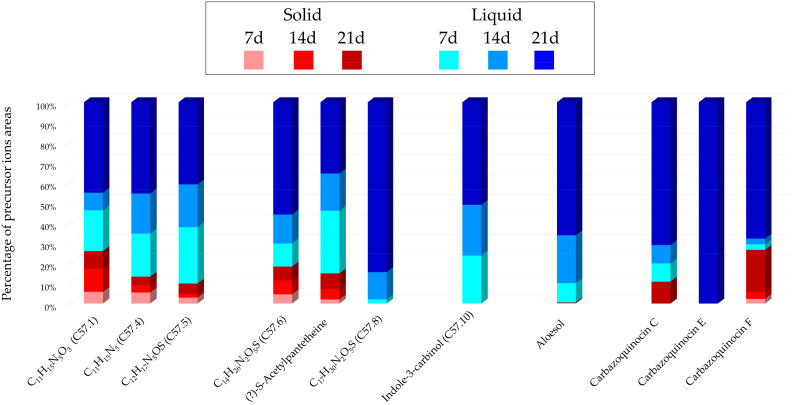
*Micromonospora* sp. SH-57: Main annotations in the IIMN designed from the extracts of the A1 solid or liquid cultures at 7, 14 and 21 days. The histograms present the cumulative proportions relative to the precursor ions areas intensities attributed to each culture condition.

Histograms in Figure 5 represent the main annotations and their proportions found for each culture condition according to the sum of precursor ion areas used for these annotations. 

The predominance of blue colors is confirmed in Figure 5, demonstrating that the majority of precursor ion areas originate from the extracts of liquid cultures. Three annotations were uniquely detected from this support, with carbazoquinocin E (C57.15, *m*/*z* 324.1958 [M + H]^+^, C_21_H_25_NO_2_) only visible in the extract from the 21-day liquid culture. This latter cultivation condition displays the highest percentages, representing a minimum of 30% to a maximum of 100% as a percentage of precursor ion areas.

##### Bioassays

Two extracts of *Micromonospora* sp. SH-57 were obtained from the solid and liquid cultures of the 14-day A1 medium and were used to evaluate the antiplasmodial and cytotoxic effects. 

**Figure 6:**
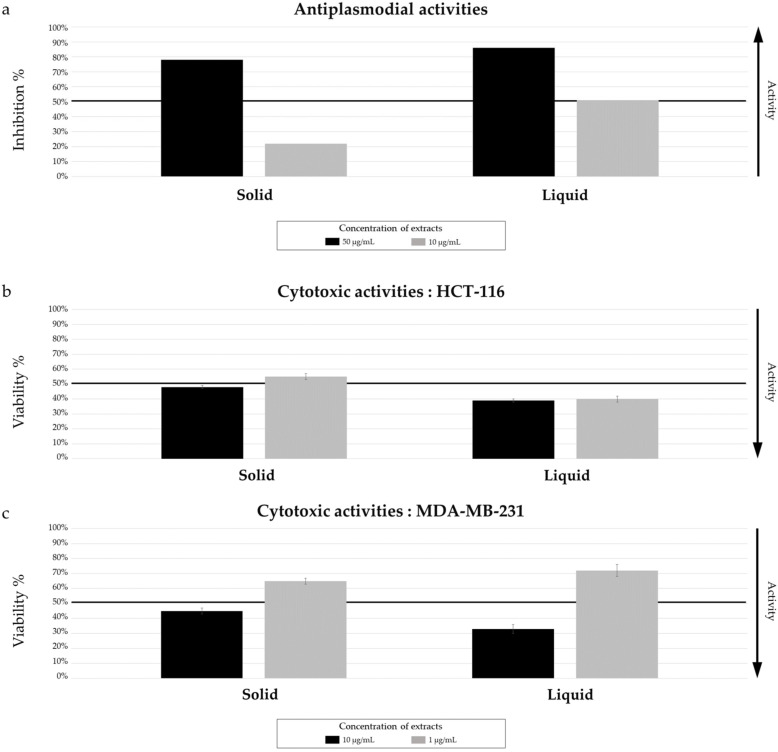
*Micromonospora* sp. SH-57: Biological activity of extracts from cultures on A1 solid and liquid media at 14 days. (**a**) Antiplasmodial activity against *P. falciparum* strain 3D7, tested at 50 µg/mL and 10 µg/mL. (**b**) Cytotoxic activity against HCT-116 cell line and (**c**) MDA-MB-231 cell line, tested at 10 µg/mL and 1 µg/mL. The black lines indicate the threshold for considering the extract as promising: antiplasmodial activity >50% inhibition; cytotoxic activity <50% viability.

If both the solid and liquid medium, extracts exhibited antiplasmodial activity higher than 50% at a concentration of 50 µg/mL. At 10 µg/mL, the extracts obtained from liquid culture demonstrated a greater inhibition percentage (over 50% versus 22%) (Figure 6a). Thus, IC_50_ was measured and gave a value of 12.06 ± 1.93 µg/mL. A slightly higher cytotoxic activity for the liquid culture extract was observed (Figure 6b,c), with a percentage viability of 40 ± 2% for the HCT-116 cell line (Figure 6b) at 1 µg/mL, indicating a promising activity.

#### 2.2.2. Influence of Medium Composition and Culture Support on *Micromonospora* sp. SH-57’s Productions

The *Micromonospora* sp. SH-57 strain was also cultivated using the two media, A1 and MB, each under solid and liquid forms, over 14 days. Four microbial crude extracts were studied. 

For these extracts, a slight increase in three visible peaks detected by HPLC-CAD was observed from the solid A1 medium compared to the solid MB medium (Table S1). Table S4, detailing the peaks, highlights the presence of a unique compound in the liquid A1 medium.

As in Figure 4, an IIMN (Figure S9a) was designed, comprising a total of 117 nodes, 38% of which were grouped into 13 clusters (>2 nodes). The blue color, picturing the extracts from A1 liquid, is predominant in the molecular network, and the diagrams in Figure S9b confirm that. Almost all nodes (94%) of the network are present from the A1 medium, and 57% are specific to it. For both media, the liquid support contains the majority (over 98%) of the nodes. The same main clusters as those represented in Figure 4c are observed in Figure S9c, with a predominance of blue and violet colors representing the liquid cultures from the two media, A1 and MB, respectively.

The annotations are presented under the form of a histogram in Figure S10, and the blue color predominates in all histograms. The A1 liquid extract constitutes over 65% of the total precursor ion areas in 8 out of the 10 presented annotations. One annotation (C57.8, *m*/*z* 375.1949 [M + H]^+^, C_17_H_30_N_2_O_5_S) was uniquely detected from liquid support extracts, and three others were specific in A1 medium extracts. 

#### 2.2.3. Overview of the Annotations

A synthesis of all the annotations obtained from the extracts of *Micromonospora* sp. SH-57 cultivated under the different conditions is presented in Table 2. Additional details and precursor ion areas for each annotation observed in MZmine software 3.6.0., according to the culture conditions provided in Table S5.

Table 2 brings out a total of 17 nodes that were successfully annotated, belonging to chemical classes, like indoles and derivatives, purine nucleoside, or benzopyrans. SIRIUS similarity scores are lower compared to *Micromonospora* sp. SH-82. Nevertheless, four annotations led to the same results with two bioinformatics tools, such as carbazoquinocin F (C 57.16, *m*/*z* 338.2113, [M + H]^+^, C_22_H_27_NO_2_), whose spectral data are provided in the appendix (Figure S11).

The investigation on the variations in intensity of the precursor ion areas (Table S5) revealed a considerable impact of two parameters: time and culture support. The observed intensities increased over time, reaching their maximum at 21 days, regardless of the support. Additionally, a rise in the intensity and number of annotations was observed for the extracts from liquid cultures compared to solid ones, regardless of the medium. The extract obtained from the A1 liquid culture at 21 days exhibited the highest intensity area for nearly all conducted annotations.

### 2.3. Salinispora arenicola SH-78

#### 2.3.1. Influence of Culture Support on *Salinispora arenicola* SH-78’s Productions

*Salinispora arenicola* SH-78 was cultivated on A1 medium under solid and liquid forms over 14 days. Two microbial crude extracts were obtained by ethyl acetate solvent extraction and analyzed to evaluate the chemical diversity. 

Seven and eight visible peaks were, respectively, detected from solid and liquid cultures after HPLC-CAD analysis (Table S1). However, the latter contains two major compounds (Table S6), among which, one is specific. 

**Figure 7:**
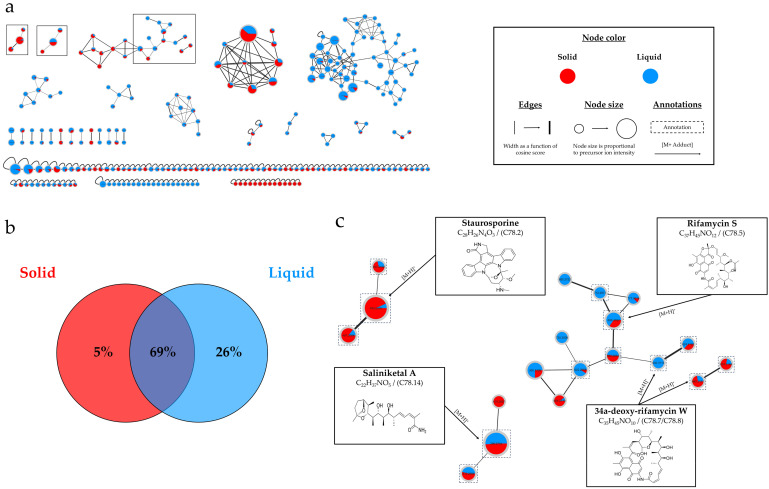
*Salinispora arenicola* SH-78: (**a**) Ion Identity Molecular Network (IIMN) from the extracts of A1 solid (red) and liquid cultures (blue) at 14 days. (**b**) Percentages of nodes as a function of culture parameter (culture support). (**c**) Zoom on 3 annotated clusters of interest.

The IIMN obtained from the extracts from the cultures of *Salinispora arenicola* SH-78 (Figure 7a) contained a total of 272 nodes, with 57% grouped into 26 clusters (>2 nodes). 

Figure 7b underscores that the majority of nodes (69%) are shared between both liquid and solid supports. However, there is an important percentage of unique nodes originating from the liquid culture (26%). In Figure 7c, the top-left cluster corresponds to the staurosporine annotations, where the red color predominates, indicating a greater presence of precursor ions from the solid culture extract. Two other interesting clusters are those of saliniketals and rifamycins families. However, the two largest clusters in the molecular network, shown in Figure S12, could not be clearly annotated, suggesting the possible presence of new metabolites. 

**Figure 8:**
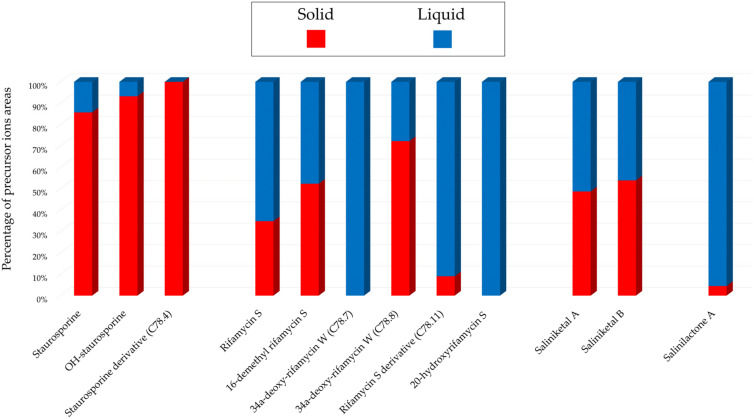
*Salinispora arenicola* SH-78: Main annotations in the IIMN designed from the extracts from A1 solid or liquid cultures at 14 days. The histograms present the cumulative proportions relative to the precursor ion areas intensities attributed to each culture condition.

In Figure 8, the histograms represent the main annotations and their proportions found for each culture condition according to the sum of precursor ion areas. 

In the middle group representing the rifamycins, higher intensities were from the extracts derived from the liquid culture. Two compounds, 34a-deoxy-rifamycin W (C78.7, *m*/*z* 640.3113 [M + H]^+^, C_35_H_45_NO_10_) and 20-hydroxyrifamycin S (C78.12, *m*/*z* 712.2971 [M + H]^+^, C_37_H_45_NO_13_), were specific to liquid cultures. Finally, the last histogram represents Salinilactone A (C78.20, *m*/*z* 183.1016 [M + H]^+^, C_10_H_14_O_3_), which was mainly observed from the liquid culture.

#### 2.3.2. Influence of Culture Time on *Salinispora arenicola* SH-78’s Productions

*Salinispora arenicola* SH-78 was also cultivated on the A1 solid medium over 7, 14, and 21 days. A total of three microbial crude ethyl acetate extracts were analyzed. 

The analyses of the HPLC-CAD chromatograms revealed a similar number of visible peaks for the three defined times, with a total of seven compounds detected (Table S1). However, a small increase in the height of the peaks was observed for the culture of 14 days compared with the other times, as shown in Table S6.

The IIMN (Figure S13a) obtained from these extracts highlighted the influence of the culture time, and it contained a total of 150 nodes, 38% of which were clustered. There is a slight dominance of the red color, representing the 14-day culture. Thus, 67% of the nodes are common to all three culture times. However, 16% are only observed at 14 days (Figure S13b). The same three clusters as in Figure 7c are observed (Figure S13c), and all nodes are present at the three times, except two exclusive rifamycin nodes at 14 days. 

Figure S14 presents the main annotations in this IIMN in histogram form. A higher proportion of precursor ions was observed in the 14-day extract, particularly for the rifamycins family, where each annotation is represented by more than 70% of the total intensity for this extract.

#### 2.3.3. Influence of Extraction Solvent on *Salinispora arenicola* SH-78’s Productions

In this part of the study, *Salinispora arenicola* SH-78 was cultivated in the solid A1 medium over 14 days. Two microbial crude extracts were obtained by successive ethyl acetate (AcOEt) and methanol (MeOH) solvent extraction. They were analyzed to evaluate their chemical diversity and biological activity.

##### Molecular Network

Figure S15a illustrates the IIMN resulting from ethyl acetate and methanol extracts and highlights the influence of the extraction solvent. The network comprises a total of 155 nodes, and 45% of them are clustered (>2 nodes).

The red color, showing the ethyl acetate extract, is predominant in the IIMN, and the diagram (Figure S15b) attests that only 38% of the nodes are common to both solvents and 45% are unique to the ethyl acetate extract. However, the main clusters of interest (Figure S15c) reveal the presence of nodes from the two extracts studied. 

Histogram representation of main annotations (Figure S16) demonstrated that 70% of precursor ion areas came from the AcOEt extract. However, within the staurosporine family, a greater presence of the precursor ion annotated as 4′-*N*-methyl-5′-hydroxy-staurosporine (C 78.3, *m*/*z* 497.2192 [M + H]^+^, C_29_H_28_N_4_O_4_) was observed in the methanolic extract.

##### Bioassays

**Figure 9:**
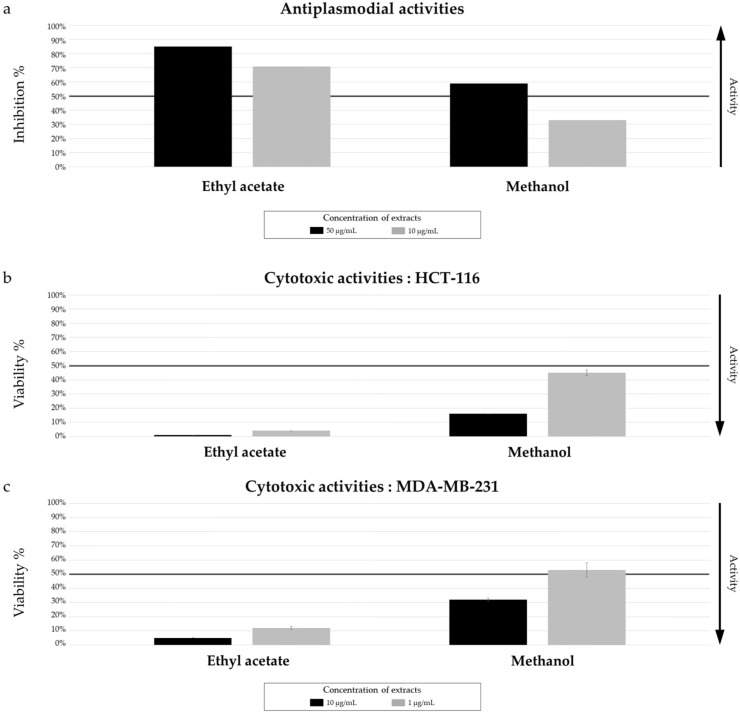
*Salinispora arenicola* SH-78: Biological activity of AcOEt and MeOH extracts from culture on A1 solid medium at 14 days. (**a**) Antiplasmodial activity against *P. falciparum* strain 3D7, tested at 50 µg/mL and 10 µg/mL. (**b**) Cytotoxic activity against HCT-116 cell line and (**c**) MDA-MB-231 cell line, tested at 10 µg/mL and 1 µg/mL. The black lines indicate the threshold for considering the extract as promising: antiplasmodial activity >50% inhibition; cytotoxic activity <50% viability.

Both ethyl-acetate and methanol extracts from the *Salinispora arenicola* SH-78’s 14-day solid cultures on A1 were evaluated for their antiplasmodial and cytotoxic activities (Figure 9).

The AcOEt extract showed 70% antiplasmodial inhibition at 10 µg/mL, while the MeOH extract was only 32% (Figure 9a). The median inhibitory concentration (IC_50_) of the AcOEt extract was evaluated at 2.57 ± 0.90 µg/mL, which is promising for a crude extract. For the cytotoxic activities, both extracts showed very promising results, with very low cell viability tested for the AcOEt extract at a concentration of 1 µg/mL (2% and 5% against HCT-116 and MDA-MB-231, respectively, in Figure 9b,c). The MeOH extract also showed promising activity at 1 µg/mL, with a viability nearly below 50% for both cell lines. 

#### 2.3.4. Overview of the Annotations 

All the annotations defined from the extracts of *Salinispora arenicola* SH-78 cultivated under the different conditions are synthesized in Table 3. Additional chemical details and precursor ion areas observed for each annotation in MZmine software, according to the culture conditions, are provided in Table S7. 

A total of 23 nodes were successfully annotated, including different chemical classes like indole derivatives or macrolactams. Seven annotations obtained the same results using at least two of the bioinformatics tools, including staurosporine (C78.2, *m*/*z* 467.2085 [M + H]^+^, C_28_H_26_N_4_O_3_) and saliniketal A (C78.14, *m*/*z* 396.2745 [M + H]^+^, C_22_H_37_NO_5_), for which the detailed spectral data are provided (Figures S17 and S18).

The salinilactones were annotated solely with the ISDB timaR tool, and the intensities were predominantly observed in the extract from liquid culture (70%) (Table S7). For the large majority of the annotations, the maximum intensity area of precursors ions observed came from the A1 liquid culture extract at 14 days. Two unique nodes (C78.7 and C78.12) were detected from this extract and were annotated as derivatives of rifamycin. Regarding the staurosporine family, higher intensities were observed in the extracts from solid cultures, with a unique ion identified as 4′-demethyl-Af-formyl-7V-hydroxy-staurosporine (C78.4, *m*/*z* 497.1825 [M + H]^+^, C_28_H_24_N_4_O_5_). 

## 3. Discussion

In order to guide a future isolation work in the quest for new molecules with therapeutic potential, this study assessed the impact of culture parameters on the metabolic diversity and biological activities of the extracts of the cultures from three preselected marine-derived actinobacteria [15,19]. The aim was to identify favorable culture parameters by varying the culture medium composition and physical state, growth duration, and nature of the solvent used for extraction. Chemical and biological results were discussed separately for each strain to highlight the specific impact of cultivation parameters; then, a transversal evaluation was carried out to identify common favorable parameters. To increase the concentration of microbial metabolites, an amberlite resin was introduced to the growth media, allowing for a more efficient capture of metabolites during the cultures [24]. Considering their relatively small amount, the biological activities detected in the crude extracts are, therefore, promising; thus, their purification could reveal more pronounced activities in the future.

### 3.1. Micromonospora sp. SH-82

- An extended cultivation time enhances the production of metabolites.

HPLC-CAD analyses showed that the culture time increased the number of compounds, especially those considered as major (>30 pA) in the extracts from the A1 medium. The number of nodes in the IIMNs increased over culture time, potentially representing an increase in the metabolic diversity. A 35% increase in the total number of nodes was observed between the extract from the A1 solid culture at 21 days compared to 7 days. For the liquid support, the increase was only +11% for the same culture durations. Ultimately, at 21 days, over 95% of the total nodes were present, regardless of the support. The raised chemical diversity related to the increase in cultivation time also seemed to correlate with an intensified biological activity. In fact, a slight increase was observed for the 21-day extract. These results demonstrated the importance of extending the culture time for *Micromonospora* sp. SH-82.

- A robust production, whatever the culture support.

In the A1 medium, the solid support appeared to enhance the production of metabolites, with higher peak intensities in HPLC-CAD analyses. The culture support shows a homogeneous distribution of nodes in the A1 medium, with each support type having a similar percentage of unique nodes (about 16%). Therefore, liquid and solid cultures seem complementary to cover a maximum of potential metabolites. However, the detailed annotations showed higher intensities from the A1 solid support compared to liquid, indicating a possible greater quantity of microbial metabolites. The impact of the culture support on biological activity was less pronounced, with a modest increase for the solid support’s extracts. The results show a good metabolite production in both liquid and solid media.

- A1 medium boosts chemical diversity, while MB medium sustains bioactivity.

In the A1 medium, the chemodiversity obtained is greater, underlined by a greater number of nodes and peak intensities, especially in the solid medium. Conversely, in the MB medium, the liquid support seems more favorable based on chemical analyses, highlighting the interplay of culture parameters among themselves. Finally, the extract derived from the MB solid medium culture, with low chemical diversity, exhibited a slightly more interesting IC_50_ than the extract from the A1 medium. This discrepancy could be explained by the relative proportions of bioactive metabolites present in the crude extract [25], the influence of the culture medium composition, or the ionization of molecules.

- Annotation of bioactive metabolites.

The annotations highlighted the presence of three major families of molecules: megalomicins, erythromycins, and erythronolides. Among the 21 annotations made, 7 were similarly obtained using the two distinct bioinformatic tools. This concordance reinforces the reliability of these annotations. Among them, four corresponded to megalomicins, a family of macrolide antibiotics isolated in 1969 from *Micromonospora megalomicea* [26]. A study conducted by Useglio et al. (2010) on the in vivo bioconversion of erythromycin C to megalomicin A [27], and the description of biosynthetic pathways involving erythromycins from the METACYC^®^ database [28], confirmed the annotations. The confluence of information, including the consensus annotations, the presence of these molecules in the biosynthetic pathways, and the former isolation from the same microbial genus, strengthens the identification of microbial metabolites.

The main annotations were visible in all the extracts from the different cultures of *Micromonospora* sp. SH-82. The extract derived from the solid A1 culture at 21 days exhibited the majority of maximal intensities, suggesting more favorable conditions for the detection of specialized metabolites. This extract also included unique annotations and numerous unannotated nodes inside the megalomicin cluster, potentially corresponding to unknown derivatives. However, in the large cluster containing erythromycins, a region mainly originates from liquid culture extracts, highlighting the importance to conduct both types of cultures to raise the chemical diversity. 

All tested extracts exhibited promising antiplasmodial activity, possibly attributed to the presence of megalomicins previously reported as active against two strains of *Plasmodium falciparum* [29]. 

### 3.2. Micromonospora sp. SH-57

- The liquid support culture is essential for the production of metabolites.

For *Micromonospora* sp. SH-57, the culture support had an important influence on the production of specialized metabolites. Chemical analyses demonstrated a preference for liquid culture in terms of chemical diversity, showing an increase in both the number and intensity of peaks (HPLC-CAD), as well as the number of nodes and precursor ion intensities, enabling annotations (HRMS-MS). The results also indicate that the solid support is unfavorable. A correlation was observed between chemical diversity and biological activities. Anticancer and antiplasmodial activities were more pronounced in the liquid medium, especially the latter.

- An extended cultivation time and A1 medium are still favorable.

As for *Micromonospora* sp. SH-82, HPLC-CAD analyses substantiated that the culture time increased the number and intensities of peaks in the extracts from *Micromonospora* sp. SH-57, particularly for the A1 liquid medium. The extract derived from this 21-day culture exhibited all the ion precursors used for the annotations, often at maximum intensities. These results confirm the influence of culture duration on metabolite production [30] and underscore a significant interest in this culture condition.

- Annotation of bioactive metabolites.

Among the annotations made, four were confirmed by the two bioinformatic tools used, including three carbazoquinocins, already isolated from actinobacteria [31]. Another annotation was aloesol, a chromone isolated from rhubarb [32], with a compound of similar structure also isolated from a *Micromonospora* sp. [33]. The annotations for the *Micromonospora* sp. SH-57 strain obtained lower identification scores and displayed a weaker correlation with the literature, compared to those conducted on the other strains. Considering the low number of potential identifications realized, the purification of compounds from this strain could lead to interesting discoveries of new structures and improve the overall network annotations. 

The extract from the 21-day liquid culture exhibited maximum intensities for nearly all annotations, with unique nodes, particularly in the carbazoquinocin cluster, highlighting these culture conditions as optimal.

Despite a low quantity of microbial metabolites, the detectable biological activities suggested potential promise for the pure molecules. The bioactive molecules annotated, such as chromones [34], carbazoquinocins [35], or purine derivatives [36], could explain the results of biological activities.

### 3.3. Salinispora arenicola SH-78

- A variable influence of time and culture support on the production of metabolites.

HPLC-CAD analyses did not show notable differences from the different solid cultures at different times. However, the results of IIMN showed an increase in the number of nodes over time, particularly from 14 days, with 98% of nodes detected compared to only 61% at 7 days. This effect is supported by the study of Crüsemann et al. (2017), which revealed an increase in chemodiversity up to 28 days for a *Salinispora arenicola* strain [37].

A small variation in the intensity of the peaks in HPLC-CAD analyses was observed in the extract from the liquid culture compared to the solid culture. The results of IIMN showed a 21% increase in the total number of nodes from the liquid culture extract, of which 26% were unique to this support. Crüsemann et al. (2017) compared 35 *Salinispora* strains on solid and liquid A1 media and also demonstrated the impact of the culture support [37]. In their study, the solid support appeared to be more favorable, and the divergence with our results could be attributed to significant interspecific peculiarities in the *Salinispora* genus [38]. In our study, the use of amberlite resin, which usually influences the production of microbial metabolites [24], could also be one of the explanations. 

Within the cluster of staurosporines, a stronger intensity of precursor ion areas is observed for the solid cultures. For the other annotations, the maximum intensity is predominantly derived from the liquid culture, as indicated by the nodes corresponding to salinilactones. These results demonstrated that the optimal parameters may vary depending on the type of targeted molecules.

- The use of different solvents increases the quantity and diversity of metabolites.

For the solid culture at 14 days, two successive solvents were used for extractions (ethyl acetate and methanol), and it was stated that the first solvent extracted the majority of metabolites. However, 55% of the nodes were also present in the second extract, with 17% being specific, illustrating the benefit of using both solvents. Crüsemann et al. (2017) showed the advantage of using three different solvents to broaden the range of extracted molecules from *Salinispora* strains [37]. The node annotated as 4′-demethyl-Af-formyl-7V-hydroxy-staurosporine exhibited its highest intensity in the methanolic extract, reinforcing the relevance of processing with different solvents.

- Annotation of bioactive metabolites.

The annotations revealed the presence of staurosporines, saliniketals, ryfamicins, and salinilactones, well known in the literature as being produced by *Salinispora* sp. [18,39,40]. Three annotations, OH-staurosporine, staurosporine, and rifamycin S, have been identified with both bioinformatics tools. The consensus annotations, the described biosynthetic pathways [41], and the literature reinforce the identification of these microbial compounds. Despite thorough investigation, the two main clusters (Figure S12) could not be clearly identified. The isolation and the identification of these compounds may be profitable due to the chemical potential of *Salinispora* strains [18]. 

The extracts tested showed remarkable biological activities, especially towards cytotoxicity. This activity may be partially attributed to the presence of staurosporine, and its derivatives are known for their anticancer activity [42,43].

### 3.4. The Common Impact of Culture Parameters on the Production of Metabolites

The present study highlighted the impact of culture and extraction parameters on the metabolic diversity and biological activities of three preselected marine-derived actinobacteria. Despite some similarities, differences have been observed between the isolates, concerning the number of presumed metabolites produced and the variations in their intensities depending on the conditions. The impact of the culture medium composition is already well known to be a major factor influencing the production of specialized metabolites [13,37]. From our study, medium A1 appears to be more conducive to obtain higher chemodiversity, at least from the two *Micromonospora* sp. isolates. Additionally, less studied parameters such as incubation time and culture support have also demonstrated considerable effects. The extension of the culture duration appeared to be a key parameter to enhance the production of specialized metabolites and is correlated with higher biological activity. However, the culture support exhibited much more specific impacts depending on the strains or types of metabolites targeted. 

## 4. Materials and Methods

### 4.1. Biological Material (Sponge/Microbial Strains)

A microbial collection of one hundred and twenty-four strains was created from the microorganisms hosted by the marine sponge *Scopalina hapalia* ML-263 [15]. The genetic identifications were achieved by the company Genoscreen, and three actinobacteria, *Micromonospora* sp. SH-82, *Micromonospora* sp. SH-57, and *Salinispora arenicola* SH-78, were selected for this study.

### 4.2. Cultivation of Strains Using the “One Strain Many Compounds” (OSMAC) Method

The OSMAC approach used in this study varied the selected culture parameters, such as incubation time (7, 14, 21 days), medium composition (A1BFe+C and Marin Broth), and support type (solid and liquid cultures). The selected strains were all kept in storage cryotubes and revived before pre-culturing, according to the protocol described in Le Loarer et al. (2023) [19]. Pre-cultures and final cultures were carried out in the same medium to obtain the crude extracts.

A1BFe+C medium, simplified to A1 in this article, consists of 10 g soluble starch (ref. 417587, BD Difco, Le Pont de Claix, France), 33 g sea salts (Instant Ocean 16 kg, Aquarium system, Sarrebourg, France), 4 g yeast extract (ref. 212750, BD Bacto, Le Pont de Claix, France), 1 g CaCO_3_ (ref. 433185, Carlo Erba, Val de Reuil, France), 2 g peptone (ref. 211820, BD Bacto, Le Pont de Claix, France), 100 mg KBr (ref. 470735, Carlo Erba, Val de Reuil, France), 40 mg Fe_2_(SO_4_)_3_ (ref. 451926, Carlo Erba, Val de Reuil, France), and QSP distilled water to obtain 1 L of final medium. Marin Broth medium, simplified to MB, was made of 30 g Marin Broth powder (ref. 279110, BD Difco, Le Pont de Claix, France) and QS distilled water for 1 L of final medium. 

Solid cultures were produced by adding 20 g agar (ref. 281210, BD Difco, Le Pont de Claix, France) to each medium, according to the protocol of Le Loarer et al. (2023) [19]. Incubation times were 7, 14, or 21 days for A1 medium and 14 days only for MB medium.

Liquid cultures were obtained by introducing 25 mL of the preculture into 500 mL of liquid A1 or MB media, which contained 25 g of sterile amberlite resin XAD-16 (ref. MFCD00145831, Sigma Aldrich, St. Louis, MO, USA). Liquid cultures were incubated at 28 °C in a thermostatically controlled incubator (ref. S-000121948, Infors FT, Bottmingen, Switzerland) under agitation speed of 180 rpm. Incubation times were the same as for solid cultures. Table 4 summarizes the main parameters applied to the different strains. These cultures were conducted in the same manner for each strain, facilitating comparisons between species. Culture controls (blanks) corresponding to the same conditions but without inoculation of the microorganisms were realized.

### 4.3. Microbial Extract Preparation

As described by Le Loarer et al. (2023) [19], after incubation, the XAD-16 amberlite resin and biomass were recovered, filtered, washed, and dried. This set was extracted for 2 h in 100 mL ethyl acetate (AcOEt) (ref. 448252 RPE grade, CarloErba, Val de Reuil, France). For *Salinispora arenicola* SH-78 culture on solid A1 medium at 14 days, a second consecutive extraction with 100 mL of methanol (MeOH) (ref. 412383 HPLC + grade, CarloErba, Val de Reuil, France) was performed during the same time. The two extracts were evaporated using a rotavapor (Laborota 4000, Heidolph, Schwabach, Germany) to obtain the microbial crude extracts. The entirety of the extracts was sent for high-resolution analyses, while a limited subset was chosen for biological activity testing, considering the quantity of available extracts.

### 4.4. Chemical Analysis

The chemical analyses carried out on the microbial extracts were similar to those in the study of Le Loarer et al. (2023) [19].

#### 4.4.1. HPLC-DAD-CAD Analysis

The microbial dry extracts were resolubilized in acetonitrile (ACN) (analytical grade 99% purity, CarloErba, Val de Reuil, France), filtered on a 0.2 µm Minisart RC filter (ref. 7764ACK, Sigma Aldrich, St. Louis, MO, USA) and standardized at a concentration of 10 mg/mL. They were analyzed by high-performance liquid chromatography (HPLC) on Dionex Ultimate 3000 (Thermo Scientific, Waltham, MA, USA) coupled to a diode array UV (DAD) and charged aerosol detector (CAD). Separation was performed on a Phenomenex Gemini C18 analytical column (150 × 4.6 mm, 3 μm) (Phenomenex, Torrance, CA, USA) with two solvents, ACN (MS grade, CarloErba, Val de Reuil, France) (phase A) and milli-Q water (phase B), each containing 0.1% formic acid (FA) (analytical-grade 99% purity, CarloErba, Val de Reuil, France). For the analysis, a linear gradient from 5 to 100% B was used for 30 min with a flow rate of 0.7 mL/min. The CAD signal provided quantitative data expressed in pA. Table S1 presents the number of visible peaks (height > 5 pA) and peaks considered as major (>30 pA), originating exclusively from microbial extracts. Tables S2, S4, and S6 provide chemical details of these peaks.

#### 4.4.2. UHPLC-QTOF-MS/MS Analysis

Standardized microbial extracts were dissolved in 1 mL of methanol (LCMS grade, CarloErba, Val de Reuil, France) and filtered with 0.22 μm PTFE syringe filters (Restek, Bellefonte, PA, USA). A Dionex Ultimate 3000 UHPLC system (Thermo Scientific, Waltham, MA, USA) coupled to a Bruker Impact II QtoF mass spectrometer (Bruker, Billerica, MA, USA) was used for high-resolution analysis. The separation was performed on a Phenomenex Kinetex phenyl hexyl analytical column (1.7 μm, 150 × 2.1 mm, 1.7 µm) (Phenomenex, Torrance, CA, USA) with two solvents, ACN (MS grade, CarloErba, Val de Reuil, France) (phase A), and milli-Q water (phase B), each containing 0.1% formic acid (FA) (analytical grade 99% purity, CarloErba, Val de Reuil, France). Elution was performed with a linear gradient from 0 to 100% B for 8 min with a flow rate of 0.5 mL/min. The main MS data acquisition parameters were as follows: an acquisition in positive mode (ESI+; 20–40 eV) and in the range of 20 to 1200 Da for MS1 spectra, a collision energy of 40 eV, an acquisition speed of 4 Hz, and the selection of the 5 major MS1 precursors for the recording of MS2 spectra.

### 4.5. Raw Data Processing, Ion Identity Molecular Networks, and Annotations

The high-resolution MS data were all processed identically with the MZmine 3.6.0 software [44]. The main processing parameters applied were as follows: a detection threshold for MS1 masses of 3^E^3 and 1^E^0 for MS2, the use of the ADAP chromatogram builder [45], and the local minimum resolver for the creation of feature tables. All parameters used for data processing are described in Table S7. The aligned feature tables and MS1/MS2 mass spectra data files (.mgf) were exported and used for the creation of Ion Identity Molecular Networks (IIMNs) and compound annotations. 

The IIMNs were obtained from files exported from MZmine 3.6.0 [44] and created using the feature-based molecular networking workflow of the GNPS platform [20,46]. The parameters set for the molecular networks are shown in Table S8. Visualization and graphical modifications were performed with Cytoscape 3.9.1 software [47]. 

Prior to annotation, the molecular networks were manually curated for each node to verify the true absence or presence of specific features in the microbial extracts. This step enabled us to obtain more robust statistics and a correction on the origin of the ion in the different samples. Feature annotation was performed by combining the results of different computational and automated pipelines, followed by manual inspection to refine and correct the feature annotation. The GNPS platform [46] was used to compare the MS2 spectra with several experimental spectral libraries, and the spectra were also compared with in silico spectral databases of natural products (ISDB) [48]. The results were refined using timaR 2.7.2 software [23] and the LOTUS database [22]. The SIRIUS 5.7.2 software (Lehrstuhl für Bioinformatik, Jena, Germany) [21] was used to predict the fragmentation patterns with CSI: fingerID module [49], the raw formula and the chemical class with the CANOPUS module [50]. The chemical classes were completed by Classyfire [51], an open access platform for identifying the chemical classes. These different tools provided different annotation scores for each feature, which were compared and grouped together if they were consistent. A similarity percentage of 100% and a score of 1 represent the highest levels of spectral similarity for the SIRIUS software, the GNPS platform, and the ISDB timaR tool, respectively. Very good confidence was assessed when the tools displayed the same annotation with a high score and when the literature was also coherent. 

### 4.6. Biological Activity Tests

Biological activity tests were performed on a selection of microbial extracts that had been produced in sufficient quantities. The antiplasmodial activity and the cytotoxic activity were targeted, and the protocols tests are detailed in Le Loarer et al. (2023) [19].

The antiplasmodial activity was carried out by the Pharmacognosy laboratory of the University of Liege using in vitro cultures of the chloroquine-sensitive 3D7 strain of *Plasmodium falciparum*, according to the procedure of Trager and Jensen [52]. Extracts showing greater than 50% inhibition at a concentration of 10 µg/mL were selected for the measurement of the concentration required to inhibit the growth of the parasite culture by 50% (IC_50_).

The cytotoxic activity was performed by the Institut de Chimie des Substances Naturelles (ICSN, Paris, France) on human colorectal HCT-116 and mammary MDA-MB-231 carcinoma cells. The percentage viability index was calculated from three experiments on extracts concentrated at 10 and 1 µg/mL. They were considered promising if the viability percentage was below 50% at a concentration of 1 µg/mL.

## 5. Conclusions

The objective of this study was to evaluate the impact of culture parameters on the metabolic diversity and biological activity of microbial extracts obtained from three marine-derived actinobacteria isolated from the marine sponge *Scopalina hapalia* (ML-263). The OSMAC method was implemented to conduct this study, varying the incubation time, culture support, and composition of the medium to obtain different microbial extracts. These extracts were subjected to chemical determination, using a quantitative analysis, the creation of Ion Identity Molecular Networks (IIMNs), and the annotation of metabolites thanks to bioinformatics tools. Biological screening was carried out by testing microbial extracts for their cytotoxic and antimalarial activities. The best culture parameters will be used for large-scale cultures, in order to isolate and further identify the attractive metabolites.

This study highlighted behavioral traits common to all strains while bringing out some specificities. Nevertheless, the parameters most favorable to enhancing the chemodiversity and the bioactivity were underscored for each isolate. Increasing the culture time up to 21 days promoted the production of metabolites and the detection of a panel of compounds from *Micromonospora* strains. These results are correlated with a raised number of nodes in the IIMNs, as well as better biological activity for the *Micromonospora* sp. SH-82 isolate. The use of an A1 medium appeared to be the most favorable to achieve a greater metabolic diversity. Notably, the *Micromonospora* sp. SH-57 strain exhibited considerably higher chemical diversity in this medium, particularly underscored by the IIMN. Finally, the influence of the culture support (liquid or solid) varied depending on the strains. *Micromonospora* sp. SH-82 was not clearly influenced in its production of metabolites by the culture supports, indicating that this microorganism maintained a robust production, regardless of the liquid or solid culture conditions. In contrast, *Salinispora arenicola* SH-78 and *Micromonospora* sp. SH-57 strains revealed a greater chemical diversity in liquid media, coupled with a higher biological activity for the latter. Undoubtedly, culture parameters have a particular impact on the production of specific metabolites, as revealed by the IIMNs. One interesting example is the solid culture extracts of *Salinispora arenicola* SH-78, which exhibited higher intensities of the compounds identified as staurosporines, already known as biologically active molecules.

This work, which assessed the impact of less explored culture parameters, such as the time of cultivation and the nature of the growth support, enabled the identification of favorable culture parameters for the *Micromonospora* and *Salinispora* marine-derived actinobacteria in the range of the studied conditions. The results highlighted the importance of systematically exploring and carefully selecting physico-chemical parameters, whether to enhance metabolic diversity or optimize the production of specialized molecules. This study could be combined with a strain selection method to choose the most promising microorganisms in their optimal production conditions. Thus, it paves the way for larger-scale cultures to isolate promising microbial metabolites for therapeutic and industrial purposes.

## Figures and Tables

**Table 1:** Summary table of annotations from the different extracts of *Micromonospora* sp. SH-82.

Compound ID	Molecular Formula	Compound Name or InChIKey ^(1,2,3)^	Chemical Class	Similarity ^(1,2,3)^
C82.1	C_44_H_80_N_2_O_15_	Megalomicin A ^(1,3)^	Organooxygen compounds	92% ^(1)^/0.05 ^(3)^
C82.2	C_45_H_78_N_2_O_17_	Megalomicin B ^(1,3)^		89% ^(1)^/0.05 ^(3)^
C82.3	C_47_H_84_N_2_O_16_	4′-Propionylmegalomicin A ^(1)^		89% ^(1)^
C82.4	C_48_H_84_N_2_O_17_	Megalomicin C1 ^(1,3)^		87% ^(1)^/0.05 ^(3)^
C82.5	C_49_H_86_N_2_O_17_	Megalomicin C2 ^(1,3)^		84% ^(1)^/0.05 ^(3)^
C82.6	C_39_H_69_NO_14_	2′-*O*-Acetylerythromycin A ^(1)^		83% ^(1)^
C82.7	C_36_H_65_NO_13_	Erythromycin C ^(1)^		98% ^(1)^
C82.8		13-Deethyl-13-methylerythromycin ^(1)^		96% ^(1)^
C82.9	C_36_H_65_NO_12_	Erythromycin D ^(1)^		93% ^(1)^
C82.10	C_37_H_67_NO_12_	Erythromycin B ^(1,3)^		91% ^(1)^/0.05 ^(3)^
C82.11	C_36_H_63_NO_12_	6-Desmethyl erythromycin D ^(1,3)^		92% ^(1)^/0.05 ^(3)^
C82.12	C_29_H_53_NO_9_	3-*O*-De(3-C,3-*O*-dimethyl-2,6-dideoxy-alpha-L-ribo-hexopyranosyl)-6-deoxyerythromycin ^(1)^		85.3% ^(1)^
C82.13				
C82.14	C_36_H_65_NO_12_	6-Deoxy-3′-*O*-demethylerythromycin ^(1)^		89% ^(1)^
C82.15	C_35_H_63_NO_13_	Norerythromycin ^(1)^		89% ^(1)^
C82.16	C_21_H_38_O_7_	Erythronolide B ^(1)^	Macrolides and analogues	62% ^(1)^
C82.17	C_20_H_36_O_7_	2-Desmethyl-2-hydroxy-6-deoxyerythronolide B ^(1)^		69% ^(1)^
C82.18	C_28_H_50_O_10_	3-*O*-Alpha-mycarosylerythronolide B ^(1)^		68% ^(1)^
C82.19	C_27_H_48_O_10_	3-*O*-(alpha-L-olivosyl)erythronolide B ^(1)^		75% ^(1)^
C82.20				75% ^(1)^
C82.21	C_21_H_38_O_6_	6-Deoxyerythronolide B ^(1,3)^		63% ^(1)^/0.05 ^(3)^

Data from ^1^ SIRIUS, ^2^ GNPS or ^3^ ISDB timaR bioinformatics tools. Each score is independent and associated with the specific workflow from which it originates.

**Table 2:** Summary table of annotations from the different extracts of *Micromonospora* sp. SH-57.

Compound ID	Molecular Formula	Compound Name or InChIKey ^(1,2,3)^	Chemical Class	Similarity ^(1,2,3)^
C57.1	C_11_H_15_N_5_O_3_	2-Deoxy-*N*6-methyladenosine ^(1)^	Purine nucleoside	93% ^(1)^
C57.2	C_11_H_15_N_5_O	SXIDRQQQIPLCTJ ^(1)^		81% ^(1)^
C57.3	C_11_H_13_N_5_O_3_	UHYRJPGYRFMFLT ^(1)^		75% ^(1)^
C57.4	C_11_H_15_N_5_	9-cyclopentyl-*N*-methylpurin-6-amine ^(1)^		75% ^(1)^
C57.5	C_12_H_17_N_5_OS	INPAYTORGXXLMB ^(1)^		66% ^(1)^
C57.6	C_14_H_26_N_2_O_5_S	JDNYVZBVEBRRCT ^(1)^	Carboxylic acids and derivatives	86% ^(1)^
C57.7	C_13_H_24_NO_5_S	(?)-*S*-Acetylpantetheine ^(1)^		77% ^(1)^
C57.8	C_17_H_30_N_2_O_5_S	ZCNIMMSEOJFZKZ ^(1)^		81% ^(1)^
C57.9	C_19_H_28_N_2_O_5_S	IXKOTSUCYPEFPP ^(1)^		78% ^(1)^
C57.10	C_9_H_9_NO	Indole-3-carbinol ^(1,2)^	Indoles and derivatives	88% ^(1)^/0.77 ^(2)^
C57.11				
C57.12	C_13_H_12_O_4_	Aloesone ^(1)^	Benzopyrans	71% ^(1)^
C57.13	C_13_H_14_O_4_	Aloesol ^(1)^		83% ^(1)^
C57.14	C_20_H_23_NO_2_	Carbazoquinocin C ^(1,3)^	Indoles and derivatives	71% ^(1)^/0.05 ^(3)^
C57.15	C_21_H_25_NO_2_	Carbazoquinocin E ^(1,3)^		61% ^(1)^/0.05 ^(3)^
C57.16	C_22_H_27_NO_2_	Carbazoquinocin F ^(1,3)^		58% ^(1)^/0.05 ^(3)^
C57.17	C_24_H_31_NO_2_	12-Carbazol-9-yldodecanoic acid ^(1)^		53% ^(1)^

Data from ^1^ SIRIUS, ^2^ GNPS or ^3^ ISDB timaR bioinformatics tools. Each score is independent and associated with the specific workflow from which it originates.

**Table 3:** Summary table of annotations from the different extracts of *Salinispora arenicola* SH-78.

Compound ID	Molecular Formula	Compound Name or InChIKey ^(1,2,3)^	Chemical Class	Similarity ^(1,2,3)^
C78.1	C_28_H_26_N_4_O_4_	OH staurosporine ^(1,2,3)^	Indoles and derivatives	85% ^(1)^/0.79 ^(2)^/0.1 ^(3)^
C78.2	C_28_H_26_N_4_O_3_	Staurosporine ^(1,2,3)^		98% ^(1)^/0.96 ^(2)^/0.1 ^(3)^
C78.3	C_29_H_28_N_4_O_4_	4′-*N*-methyl-5′-hydroxy-staurosporine ^(1)^		78% ^(1)^
C78.4	C_28_H_24_N_4_O_5_	4′-demethyl-Af-formyl-7V-hydroxy-staurosporine ^(1)^		74% ^(1)^
C78.5	C_37_H_45_NO_12_	Rifamycin S ^(1,2,3)^	Macrolactams	75% ^(1)^/0.74 ^(2)^/0.05 ^(3)^
C78.6	C_36_H_43_NO_12_	16-demethyl rifamycin S ^(3)^		0.1 ^(3)^
C78.7	C_35_H_45_NO_10_	34a-deoxy-rifamycin W ^(1)^		62% ^(1)^
C78.8				58% ^(1)^
C78.9	C_34_H_41_NO_10_	Proansamycin B ^(1,3)^		64% ^(1)^/0.05 ^(3)^
C78.10				54%^(1)^
C78.11	C_34_H_41_NO_11_	Demethyl-desacetyl-rifamycin S ^(1)^		71% ^(1)^
C78.12	C_37_H_45_NO_13_	20-hydroxyrifamycin S ^(1,3)^		74% ^(1)^/0.05 ^(3)^
C78.13	C_35_H_42_NO_12_	30-hydroxyrifamycin W ^(1)^		59% ^(1)^
C78.14	C_22_H_37_NO_5_	Saliniketal A ^(1,3)^	Prenol lipids	43% ^(1)^/0.05 ^(3)^
C78.15	C_22_H_37_NO_6_	Saliniketal B ^(1,3)^		49% ^(1)^/0.13 ^(3)^
C78.16	C_8_H_10_O_3_	Salinilactone D ^(3)^	Lactones	0.13 ^(3)^
C78.17	C_9_H_12_O_3_	Salinilactone E ^(3)^		0.13 ^(3)^
C78.18				0.13 ^(3)^
C78.19	C_10_H_14_O_3_	Salinilactone A ^(3)^		0.13 ^(3)^
C78.20				0.13 ^(3)^
C78.21				0.13 ^(3)^
C78.22	C_11_H_16_O_3_	Salinilactone C ^(3)^		0.13 ^(3)^
C78.23	C_12_H_18_O_3_	Salinilactone H ^(3)^		0.13 ^(3)^

Data from ^1^ SIRIUS, ^2^ GNPS or ^3^ ISDB timaR bioinformatics tools. Each score is independent and associated with the specific workflow from which it originates.

**Table 4:** Culture parameters studied for the three actinobacteria.

Microbial Strains	Days	Support	Culture Medium
*Micromonospora* sp. (SH-82 and SH-57)	7/14/21	Solid/Liquid	A1BFe+C
	14		MB
*Salinispora arenicola* SH-78	7/14/21	Solid	A1BFe+C
	14	Liquid	

## Data Availability

The molecular networks produced for this study are available with these IDs. IIMN of *Micromonospora* sp. SH-82: 820b3e2ec42546b8bd9d4da0aab3d1d0; c57e3cafc0294e1497a7d603a25efb50. IIMN of *Micromonospora* sp. SH-57: b22f71bb29d5409c9547335e72b58e3b; cb81ad26159b41578d6e7ea860f07522. IIMN of *Salinispora arenicola* SH-78: 1917bc9b3a50493b8183febb2fe7e608; 6b57aa3a965543a18c0e5132b73562fc; 7582705fe7fe49009b1b7a7ef3cad727. High-resolution raw extract data can be supplied by e-mail.

## Supplementary Materials

The following supporting information can be downloaded at: https://www.mdpi.com/article/10.3390/md22010023/s1, Table S1. Summary table of number of peaks (visible and major) observed for each microbial extract in HPLC-CAD analysis. Table S2. Detailed of observed peaks in the HPLC-CAD chromatographic profiles of microbials extracts from *Micromonospora* sp. SH-82. Table S3. Summary table of annotations from the Ion Identity Molecular Network of the different extracts of *Micromonospora* sp. SH-82. Table S4. Detailed of observed peaks in the HPLC-CAD chromatographic profiles of microbials extracts from *Micromonospora* sp. SH-57. Table S5. Summary table of annotations from the Ion Identity Molecular Network of the different extracts of *Micromonospora* sp. SH-57. Table S6. Detailed of observed peaks in the HPLC-CAD chromatographic profiles of microbials extracts from *Salinispora arenicola* SH-78. Table S7. Summary table of annotations from the Ion Identity Molecular Network of the different extracts of *Salinispora arenicola* SH-78. Table S8. Batch mode use for data processing with MZmine 3. Table S9. Parameters use for molecular network with GNPS. Figure S1. *Micromonospora* sp. SH-82: (a) Ion Identity Molecular Network (IIMN) from the extracts of A1 solid (red) and liquid cultures (blue), and the extracts of MB solid (orange) and liquid cultures (purple), at 14 days. (b) Percentages of nodes as a function of culture parameters (culture medium and support). (c) Zoom on 3 annotated clusters of interest. Figure S2. *Micromonospora* sp. SH-82: Main annotations in the IIMN designed from the extracts of A1 or MB solid and liquid cultures at 14 days. The histograms present the cumulative proportions relative to the precursor ion areas intensities attributed to each culture condition. Figure S3. *Micromonospora* sp. SH-82: Biological activity of extracts from cultures on A1 and MB solid medium at 14 days. (a) Antiplasmodial activity against *P. falciparum* strain 3D7, tested at 50 µg/mL and 10 µg/mL. (b) Cytotoxic activity against HCT-116 cell line and (c) MDA-MB-231 cell line, tested at 10 µg/mL and 1 µg/mL. The black lines indicate the threshold for considering the extract as promising: antiplasmodial activity >50% inhibition; cytotoxic activity <50% viability. Figure S4. Spectral data of megalomicin C1 (C 82.4, *m*/*z* 961.5883 [M + H]^+^, C_48_H_84_N_2_O_17_): (a) MS1 and (b) MS2. Figure S5. Spectral data of erythromycin B (C 82.10, *m*/*z* 718.4717 [M + H]^+^, C_37_H_67_NO_12_): (a) MS1 and (b) MS2. Figure S6. Spectral data of 6-deoxyerythronolide B (C 82.10, *m*/*z* 718.4717 [M + H]^+^, C_37_H_67_NO_12_): (a) MS1 and (b) MS2. Figure S7. Cluster of *Micromonospora* sp. SH-82 containing discharged ions representing the megalomicins. Figure S8. Zoom-in on cluster of *Micromonospora* sp. SH-82 containing erythromycin annotation. Figure S9. *Micromonospora* sp. SH-57: (a) Ion Identity Molecular Network (IIMN) from the extracts of A1 solid (red) and liquid cultures (blue), and the extracts of MB solid (orange) and liquid cultures (purple), at 14 days. (b) Percentages of nodes as a function of culture parameters (culture medium and support). (c) Zoom on 3 annotated clusters of interest. Figure S10. *Micromonospora* sp. SH-57: Main annotations in the IIMN designed from the extracts of A1 or MB solid and liquid cultures at 14 days. The histograms present the cumulative proportions relative to the precursor ion areas intensities attributed to each culture condition. Figure S11. Spectral data of carbazoquinocin F (C 57.16, *m*/*z* 338.2113, [M + H]^+^, C_22_H_27_NO_2_): (a) MS1 and (b) MS2. Figure S12. Zoom-in on two largest cluster of *Salinispora arenicola* SH-78 is not clearly annotated. Figure S13. *Salinispora arenicola* SH-78: (a) Ion Identity Molecular Network (IIMN) from the extracts of A1 solid cultures (red gradient) at 7, 14 and 21 days. (b) Percentages of nodes as a function of duration. (c) Zoom on 3 annotated clusters of interest. Figure S14. *Salinispora arenicola* SH-78: Main annotations in the IIMN designed from the extracts of A1 solid cultures at 7, 14 and 21 days. The histograms present the cumulative proportions relative to the precursor ion areas intensities attributed to each culture condition. Figure S15. *Salinispora arenicola* SH-78: (a) Ion Identity Molecular Network (IIMN) from AcOEt (red) and MeOH extracts of the A1 solid medium culture at 14 days. (b) Percentages of nodes as a function of extractions’ solvent. (c) Zoom on 3 annotated clusters of interest. Figure S16. *Salinispora arenicola* SH-78: Main annotations in the IIMN designed from AcOEt and MeOH extracts from the A1 solid medium culture at 14 days. The histograms present the cumulative proportions relative to the precursor ion areas intensities attributed to each extraction condition. Figure S17. Spectral data of staurosporine (C78.2, *m*/*z* 467.2085 [M + H]^+^, C_28_H_26_N_4_O_3_): (a) MS1 and (b) MS2. Figure S18. Spectral data of saliniketal A (C78.14, m/z 396.2745 [M + H]+, C_22_H_37_NO_5_): (a) MS1 and (b) MS2.
