# Supplementary material for: OSMAC Method to Assess Impact of Culture Parameters on Metabolomic Diversity and Biological Activity of Marine-Derived Actinobacteria
Source: Mar Drugs. 2023 Dec 28;22(1):23. doi: 10.3390/md22010023 (PMC10817652; doi:10.3390/md22010023)
Supplement: Supplementary file 1 [file marinedrugs-22-00023-s001.zip › marinedrugs-2642408-supplementary.pdf]

Table S1. Summary table of number of peaks (visible and major) observed for each microbial extract in HPLC-CAD analysis.

Microbial strain	Code extract	Culture conditions			Number of peaks detected by HPLC-CAD	
		Medium	Support	Days	Visible peaks (Height > 5 pA)	Major peaks (Height > 30 pA)
Micromonospora sp. SH-82	82 A1 S 7 Ac	A1	Solid	7	13	2
	82 A1 S 14 Ac			14	19	9
	82 A1 S 21 Ac			21	21	12
	82 A1 L 7 Ac		Liquid	7	14	5
	82 A1 L 14 Ac			14	20	5
	82 A1 L 21 Ac			21	22	7
	82 MB S 14 Ac	MB	Solid	14	10	1
	82 MB L 14 Ac		Liquid	14	16	10
Micromonospora sp. SH-57	57 A1 S 7 Ac	A1	Solid	7	4	0
	57 A1 S 14 Ac			14	4	0
	57 A1 S 21 Ac			21	6	1
	57 A1 L 7 Ac		Liquid	7	4	0
	57 A1 L 14 Ac			14	4	0
	57 A1 L 21 Ac			21	10	2
	57 MB S 14 Ac	MB	Solid	14	1	0
	57 MB L 14 Ac		Liquid	14	3	0
Salinispora arenicola SH-78	78 A1 S 7 Ac	A1	Solid	7	7	0
	78 A1 S 14 Ac			14	7	0
	78 A1 S 21 Ac			21	7	0
	78 A1 L 14 Ac		Liquid	14	8	2

Table S2. Detailed of observed peaks in the HPLC-CAD chromatographic profiles of microbials extracts from *Micromonospora* sp. SH-82.

**Culture conditions**								
**Medium**	A1						MB	
**Support**	Solid			Liquid			Solid	Liquid
**Days**	7	14	21	7	14	21	14	14
**Code extract**	82 A1 S 7 Ac	82 A1 S 14 Ac	82 A1 S 21 Ac	82 A1 L 7 Ac	82 A1 L 14 Ac	82 A1 L 21 Ac	82 MB S 14 Ac	82 MB L 14 Ac

**Peaks code**	**Retention Time (min)**	**Height of peaks (pA)**							
		**82 A1 S 7 Ac**	**82 A1 S 14 Ac**	**82 A1 S 21 Ac**	**82 A1 L 7 Ac**	**82 A1 L 14 Ac**	**82 A1 L 21 Ac**	**82 MB S 14 Ac**	**82 MB L 14 Ac**
P 82.1	10.0	-	17	32	-	-	-	-	8
P 82.2	10.8	-	48	64	-	-	-	-	21
P 82.3	11.0	-	23	34	-	-	-	-	38
P 82.4	11.5	17	38	47	10	10	11	-	31
P 82.5	11.8	-	73	91	46	50	52	8	45
P 82.6	12.3	20	44	60	45	46	46	5	42
P 82.7	13.0	11	15	15	20	20	24	-	-
P 82.8	14.1	8	25	26	9	9	8	-	-
P 82.9	15.4	6	23	13	8	8	7	-	-
P 82.10	19.0	75	149	187	37	59	115	39	114
P 82.11	19.3	-	17	17	5	7	7	-	11
P 82.12	20.7	29	63	81	46	64	60	11	44
P 82.13	21.5	7	28	30	26	27	20	5	30
P 82.14	21.7	8	25	28	20	30	29	-	12
P 82.15	22.8	54	114	132	55	60	57	26	62
P 82.16	24.1	-	5	6	7	9	7	-	-
P 82.17	24.7	-	-	-	8	18	15	-	-
P 82.18	25.0	-	-	-	-	7	6	-	-
P 82.19	25.3	-	-	7	-	7	6	9	8
P 82.20	26.4	-	-	-	-	9	11	-	-

Table S2. *Continued*

**Culture conditions**									
**Medium**		A1						MB	
**Support**		Solid			Liquid			Solid	Liquid
**Days**		7	14	21	7	14	21	14	14
**Code extract**		82 A1 S 7 Ac	82 A1 S 14 Ac	82 A1 S 21 Ac	82 A1 L 7 Ac	82 A1 L 14 Ac	82 A1 L 21 Ac	82 MB S 14 Ac	82 MB L 14 Ac
**Peaks code**	**Retention Time (min)**	**Height of peaks (pA)**							
		**82 A1 S 7 Ac**	**82 A1 S 14 Ac**	**82 A1 S 21 Ac**	**82 A1 L 7 Ac**	**82 A1 L 14 Ac**	**82 A1 L 21 Ac**	**82 MB S 14 Ac**	**82 MB L 14 Ac**
P 82.21	27.1	-	-	-	-	6	6	-	-
P 82.22	27.5	-	-	-	-	5	7	-	-
P 82.23	32.7	-	-	15	-	-	-	15	-
P 82.24	33.2	9	10	14	-	-	7	-	8
P 82.25	34.7	8	49	48	-	7	34	11	50
P 82.26	35.3	14	66	72	-	5	32	45	37

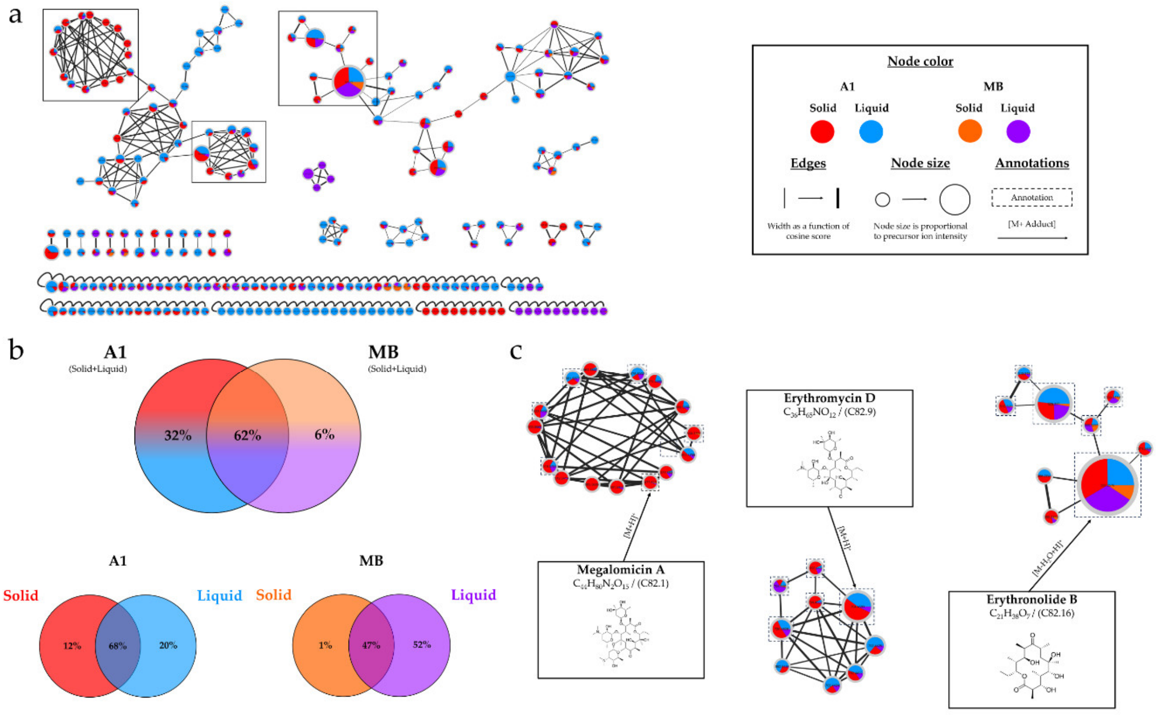

Figure S1. *Micromonospora* sp. SH-82 : Identity Molecular Network (IIMN) from the extracts of A1 solid (red) and liquid cultures (blue), and the extracts of MB solid (orange) and liquid cultures (purple), at 14 days. (b) Percentages of nodes as a function of culture parameters (culture medium and support). (c) Zoom on 3 annotated clusters of interest.

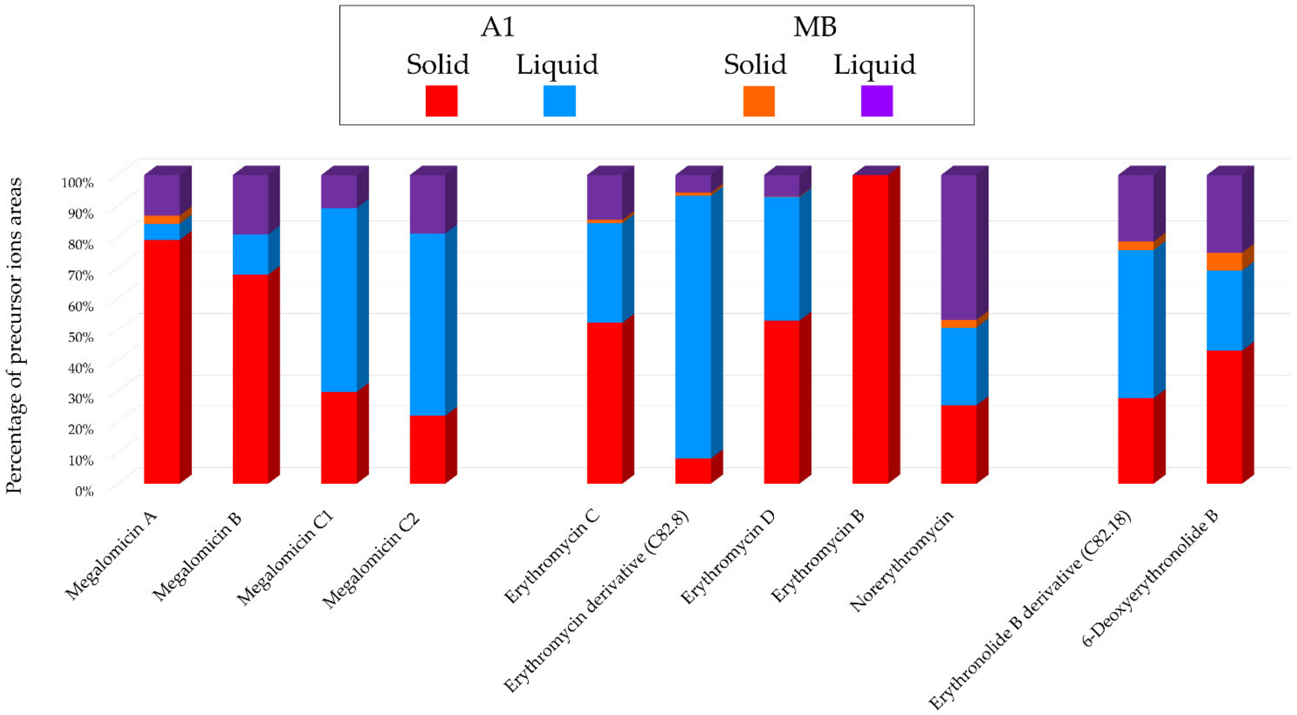

Figure S2. *Micromonospora* sp. SH-82 : Main annotations in the IIMN designed from the extracts of A1 or MB solid and liquid cultures at 14 days. The histograms present the cumulative proportions relative to the precursor ions areas intensities attributed to each culture condition.

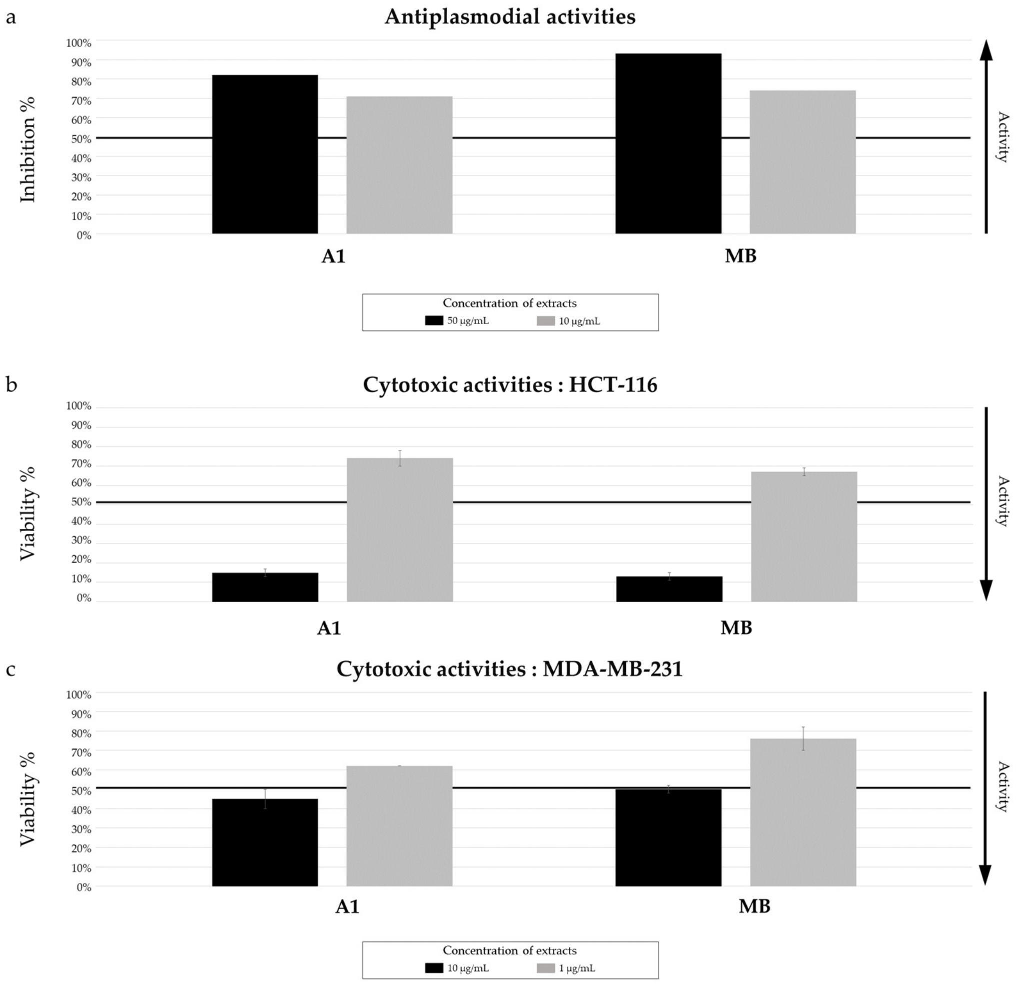

Figure S3. *Micromonospora* sp. SH-82 : Biological activity of extracts from cultures on A1 and MB solid medium at 14 days. (a) Antiplasmodial activity against *P. falciparum* strain 3D7, tested at 50 µg/mL and 10 µg/mL. (b) Cytotoxic activity against HCT-116 cell line and (c) MDA-MB-231 cell line, tested at 10 µg/mL and 1 µg/mL. The black lines indicate the threshold for considering the extract as promising: antiplasmodial activity > 50% inhibition; cytotoxic activity < 50% viability.

Table S3. Summary table of annotations from the Ion Identity Molecular Network of the different extracts of *Micromonospora sp.* SH-82.

Compound ID	RT	m/z [+Adduct]	Compound name or InChIKey (1,2,3)	Precursor ions areas observed in MzMine (with the maximum in bold) according to the culture conditions								
				**Medium**	**A1**						**MB**	
				**Support**	**Solid**			**Liquid**			**Solid**	**Liquid**
				**Days**	**7**	**14**	**21**	**7**	**14**	**21**	**14**	
C82.1	6,14	877,5648 $[M+H]^+$	Megalomicin A (1,3)		2,0E+02	3,0E+03	**6,6E+03**	1,0E+02	2,0E+02	2,0E+02	1,0E+02	5,0E+02
C82.2	6,34	919,5754 $[M+H]^+$	Megalomicin B (1,3)		1,4E+02	5,2E+03	**8,1E+03**	2,0E+02	1,0E+03	1,0E+03	1,0E+02	5,0E+02
C82.3	6,52	933,5939 $[M+H]^+$	4′-Propionylmegalomicin A (1)		1,0E+02	1,3E+03	**2,2E+03**	1,0E+02	3,0E+02	3,0E+02	-	6,0E+02
C82.4	6,59	961,5883 $[M+H]^+$	Megalomicin C1 (1,3)		1,0E+02	3,5E+03	5,7E+03	3,3E+03	**7,1E+03**	6,7E+03	-	1,3E+03
C82.5	6,77	975,6046 $[M+H]^+$	Megalomicin C2 (1,3)		1,0E+02	1,2E+03	1,7E+03	1,4E+03	3,4E+03	**3,5E+03**	-	1,1E+03
C82.6	7,26	776,4797 $[M+H]^+$	2′-*O*-Acetylerythromycin A (1)		5,0E+02	8,0E+03	5,9E+03	6,0E+03	9,8E+03	**1,2E+04**	5,0E+01	4,2E+03
C82.7	6,75	720,4529 $[M+H]^+$	Erythromycin C (1)		6,7E+03	4,8E+04	**4,9E+04**	1,8E+04	3,0E+04	2,7E+04	1,0E+03	1,3E+04
C82.8	7,24	720,4536 $[M+H]^+$	13-Deethyl-13-methylerythromycin (1)		4,8E+03	4,2E+03	2,0E+03	2,1E+04	**4,5E+04**	2,8E+04	5,0E+02	3,0E+03
C82.9	7,08	704,4586 $[M+H]^+$	Erythromycin D (1)		6,0E+03	1,3E+05	**1,4E+05**	6,8E+04	9,5E+04	7,0E+04	5,0E+02	1,7E+04
C82.10	7,25	718,4717 $[M+H]^+$	Erythromycin B (1,3)		-	1,5E+03	**3,2E+03**	-	-	-	-	-
C82.11	6,98	690,4431 $[M+H]^+$	6-Desmethyl erythromycin D (1,3)		7,3E+02	**4,3E+03**	3,7E+03	1,0E+03	5,0E+02	5,0E+02	1,0E+02	1,0E+03
C82.12	6,92	560,3782 $[M+H]^+$	3-*O*-De(3-C,3-*O*-dimethyl-2,6-dideoxy-alpha-L-ribo-hexopyranosyl)-6-deoxyerythromycin (1)		-	3,6E+03	**5,5E+03**	-	-	-	2,0E+02	-
C82.13	6,72				-	8,0E+02	**1,0E+03**	-	-	-	-	-
C82.14	7,35	704,4521 $[M+H]^+$	6-Deoxy-3'-*O*-demethylerythromycin (1)		1,0E+02	1,3E+03	1,6E+03	**2,0E+03**	1,0E+02	1,0E+02	1,0E+01	5,0E+01
C82.15	6,67	706,4521 $[M+H]^+$	Norerythromycin (1)		5,0E+02	1,0E+03	5,0E+02	5,0E+02	1,0E+03	1,4E+03	1,0E+02	**1,9E+03**
C82.16	7,54	385,2577 $[M-H_2O+H]^+$	Erythronolide B (1)		7,9E+04	**>2E+05***	**>2E+05***	8,0E+04	1,7E+05	**>2E+05***	6,2E+04	**>2E+05***
C82.17	7,30	371,2423 $[M-H_2O+H]^+$	2-Desmethyl-2-hydroxy-6-deoxyerythronolide B (1)		1,0E+02	**1,9E+03**	1,1E+03	2,0E+02	7,0E+02	5,0E+02	5,0E+01	2,0E+02
C82.18	7,99	529,3367 $[M-H_2O+H]^+$	3-*O*-Alpha-mycarosylerythronolide B (1)		1,5E+04	8,8E+04	1,3E+05	6,7E+04	**1,5E+05**	1,5E+05	8,8E+03	6,9E+04
C82.19	7,71	515,3209 $[M-H_2O+H]^+$	3-*O*-(alpha-L-olivosyl)erythronolide B (1)		1,7E+03	1,2E+04	**1,6E+04**	4,2E+03	7,9E+03	7,3E+03	5,0E+02	4,3E+03
C82.20	7,89	515,3183 $[M-H_2O+H]^+$			5,0E+02	1,1E+03	**2,0E+03**	5,0E+02	1,7E+03	2,0E+03	5,0E+01	3,0E+02
C82.21	8,25	369,2626 $[M-H_2O+H]^+$	6-Deoxyerythronolide B (1,3)		2,5E+04	1,2E+05	1,4E+05	5,3E+04	6,9E+04	**7,3E+04**	1,5E+04	6,7E+04

Data from [1] SIRIUS, [2] GNPS or [3] ISDB timaR bioinformatics tools.
*saturated values

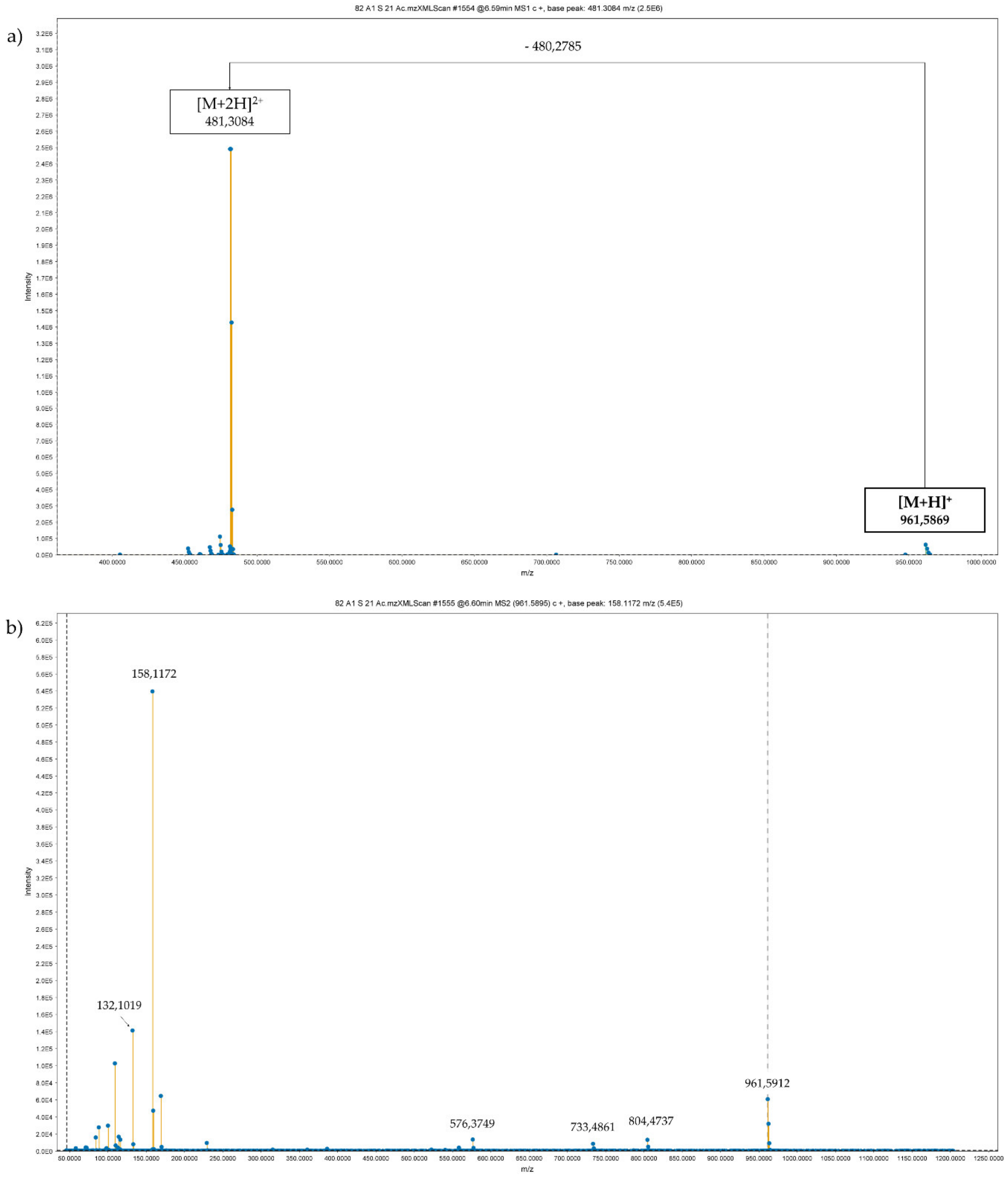

Figure S4. Spectral data of megalomicin C1
(C 82.4, *m*/z 961.5883 [M+H]$^+$, $C_{48}H_{84}N_2O_{17}$) : (a) MS1 and (b) MS2.

a)

b)

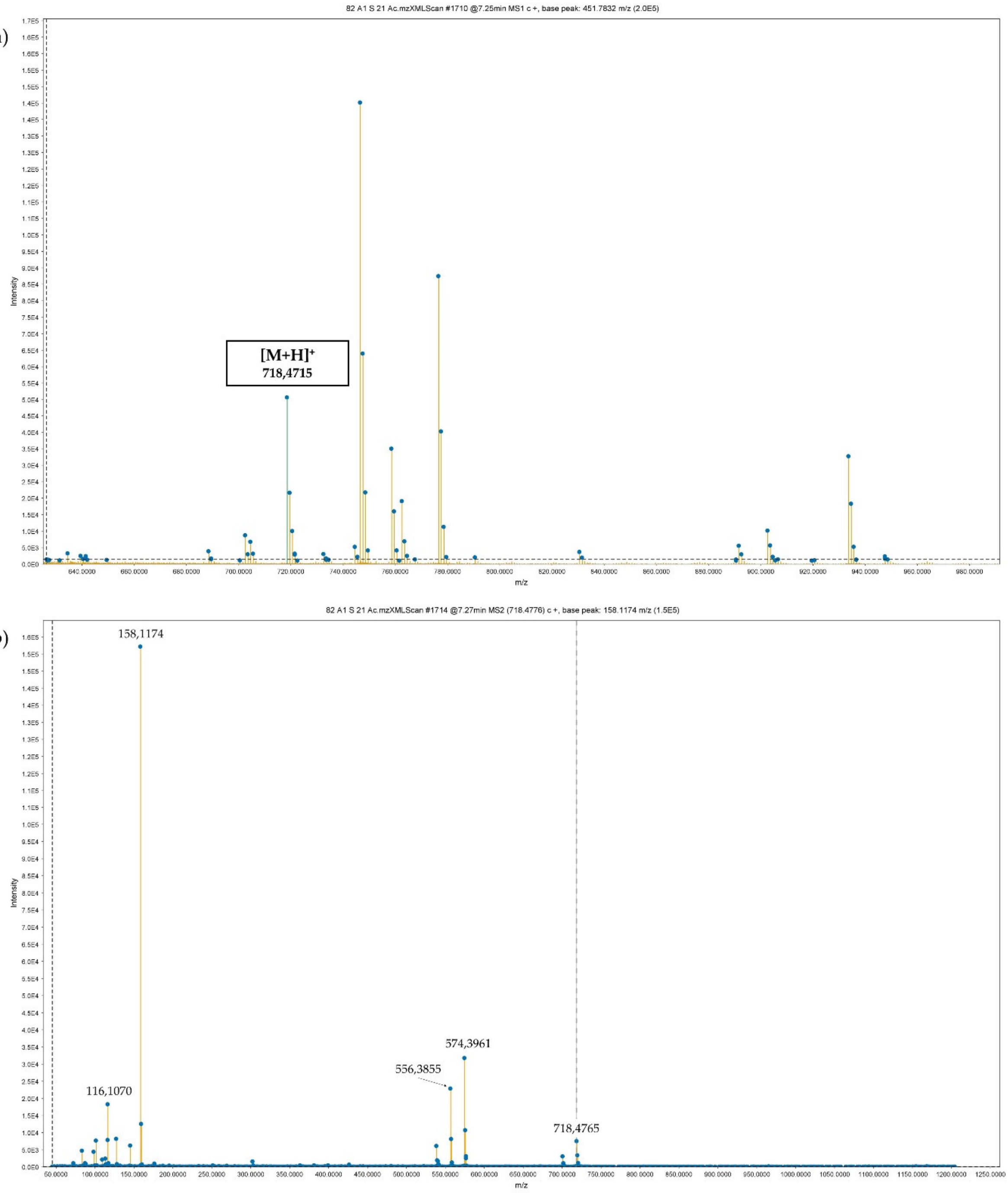

Figure S5. Spectral data of erythromycin B (C 82.10, *m*/z 718.4717 $[M+H]^+$, $C_{37}H_{67}NO_{12}$) : (a) MS1 and (b) MS2.

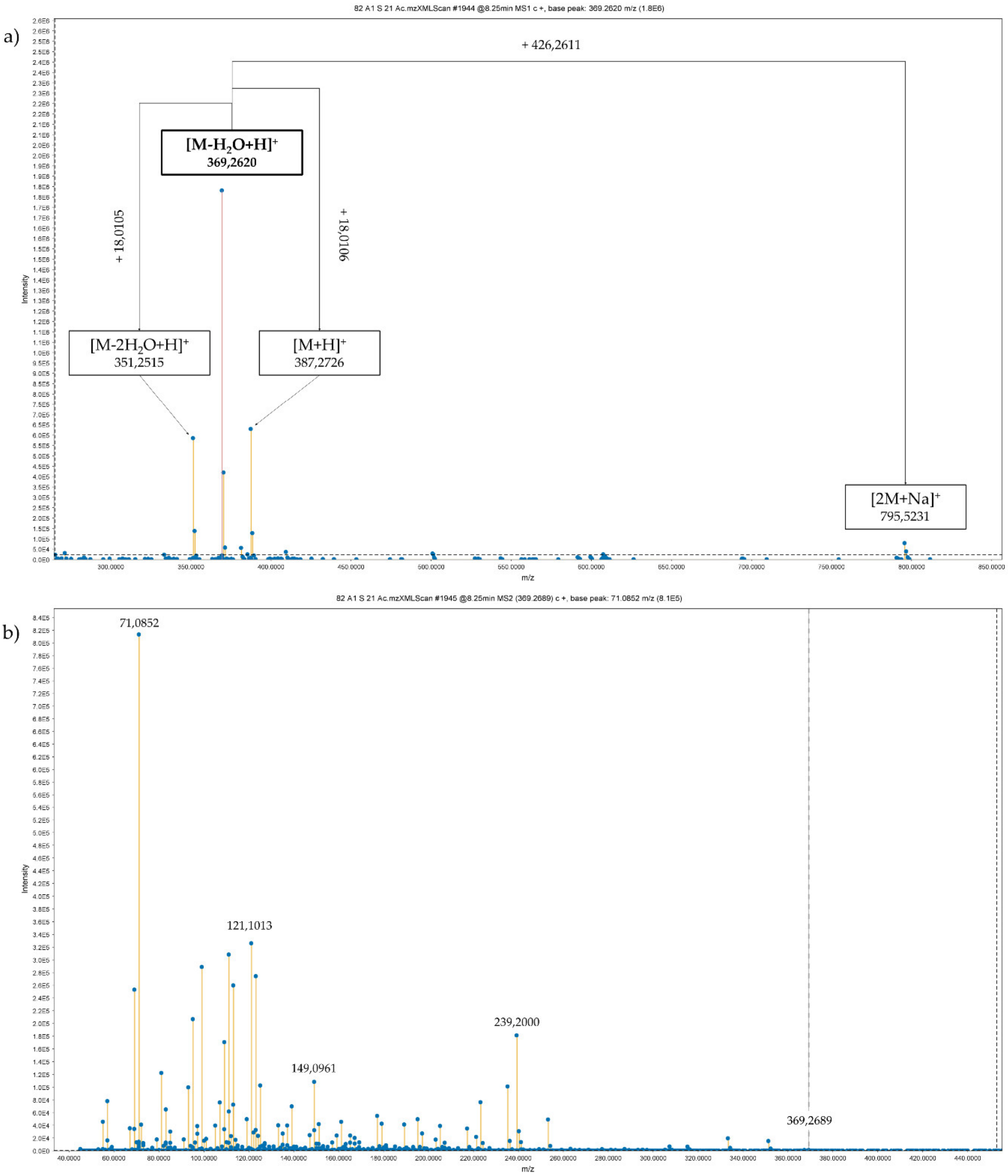

Figure S6. Spectral data of 6-deoxyerythronolide B (C 82.10, *m*/z 718.4717 [M+H]+, $C_{37}H_{67}NO_{12}$) : (a) MS1 and (b) MS2.

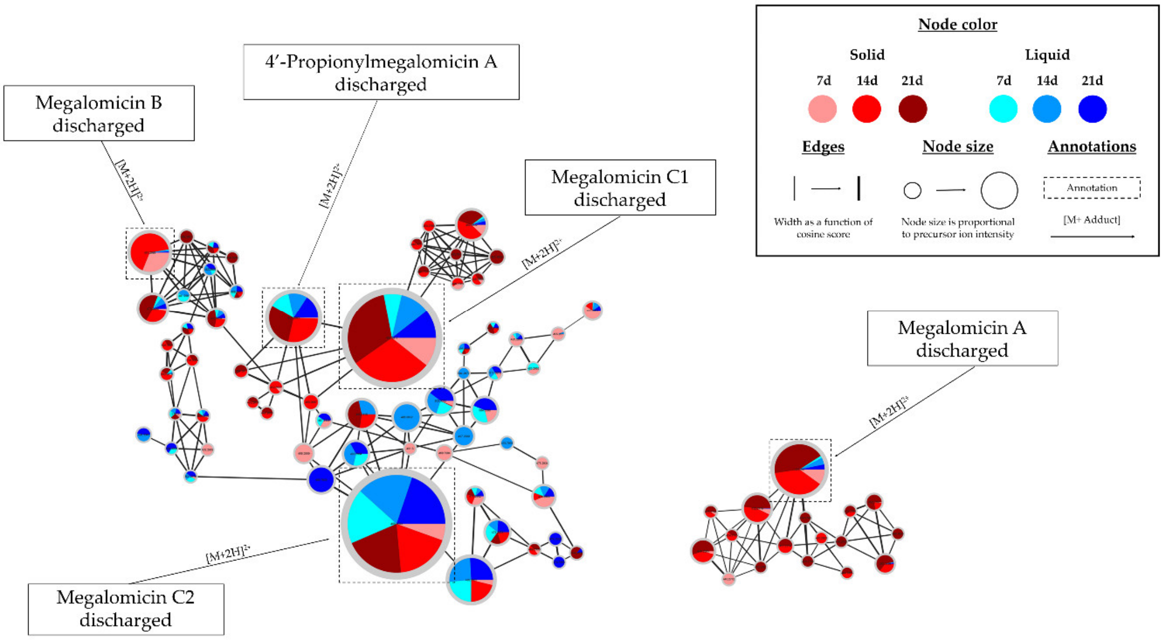

Figure S7. Cluster of *Micromonospora* sp. SH-82 containing discharged ions representing the megalomicins.

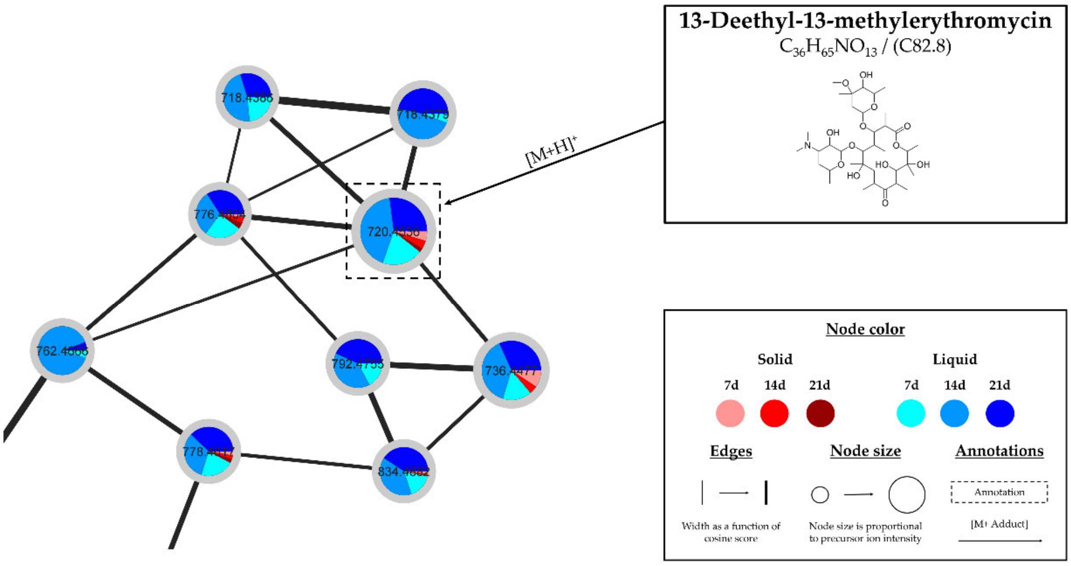

Figure S8. Zoom-in on cluster of *Micromonospora* sp. SH-82 containing erythromycin annotation.

Table S4. Detailed of observed peaks in the HPLC-CAD chromatographic profiles of microbials extracts from *Micromonospora* sp. SH-57.

**Culture conditions**									
**Medium**		A1						MB	
**Support**		Solid			Liquid			Solid	Liquid
**Days**		7	14	21	7	14	21	14	14
**Code extract**		57 A1 S 7 Ac	57 A1 S 14 Ac	57 A1 S 21 Ac	57 A1 L 7 Ac	57 A1 L 14 Ac	57 A1 L 21 Ac	57 MB S 14 Ac	57 MB L 14 Ac
**Peaks code**	**Retention Time (min)**	**Height of peaks (pA)**							
		**57 A1 S 7 Ac**	**57 A1 S 14 Ac**	**57 A1 S 21 Ac**	**57 A1 L 7 Ac**	**57 A1 L 14 Ac**	**57 A1 L 21 Ac**	**57 MB S 14 Ac**	**57 MB L 14 Ac**
P 57.1	12.2	-	-	6	12	11	8	-	10
P 57.2	16.4	-	-	-	10	41	6	-	-
P 57.3	26.7	-	-	-	-	-	5	-	-
P 57.4	26.9	-	-	-	-	-	5	-	-
P 57.5	31.4	9	5	16	15	19	20	-	21
P 57.6	34.4	13	13	16	-	-	17	8	-
P 57.7	35.3	22	17	32	6	6	43	-	5
P 57.8	36.4	12	18	25	-	-	36	-	-
P 57.9	37.6	-	-	-	-	-	9	-	-
P 57.10	39.1	-	-	7	-	-	24	-	-

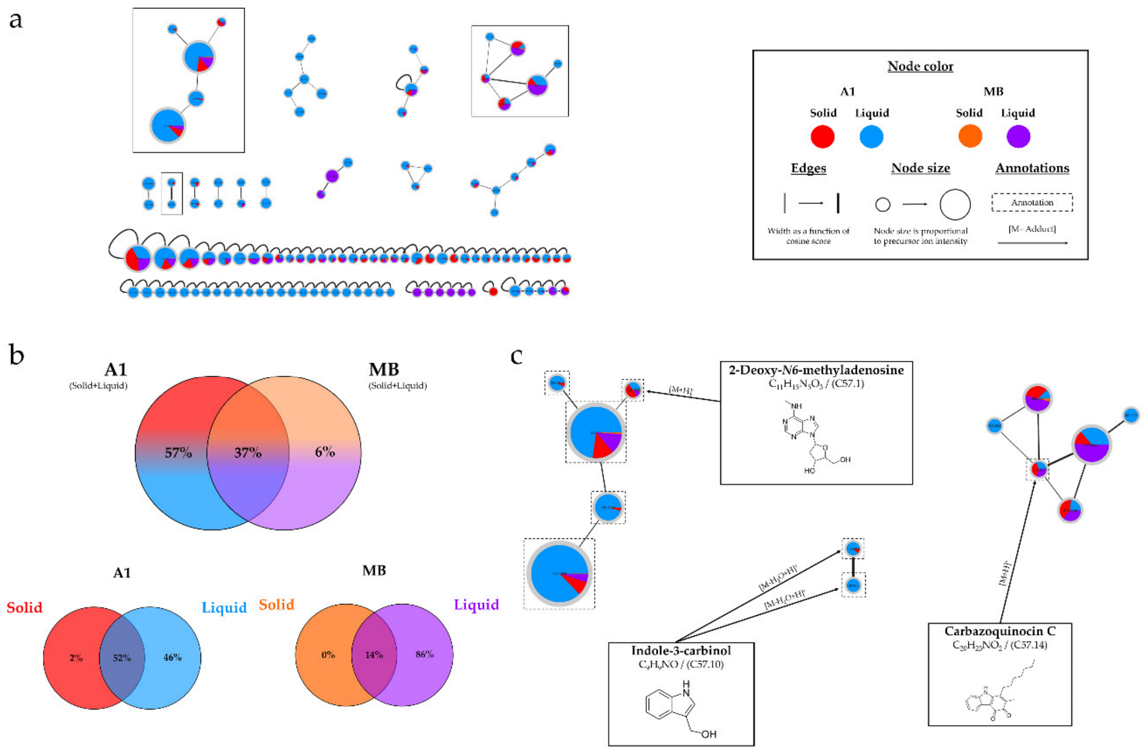

Figure S9. *Micromonospora* sp. SH-57 : (a) Ion Identity Molecular Network (IIMN) from the extracts of A1 solid (red) and liquid cultures (blue), and the extracts of MB solid (orange) and liquid cultures (purple), at 14 days. (b) Percentages of nodes as a function of culture parameters (culture medium and support). (c) Zoom on 3 annotated clusters of interest.

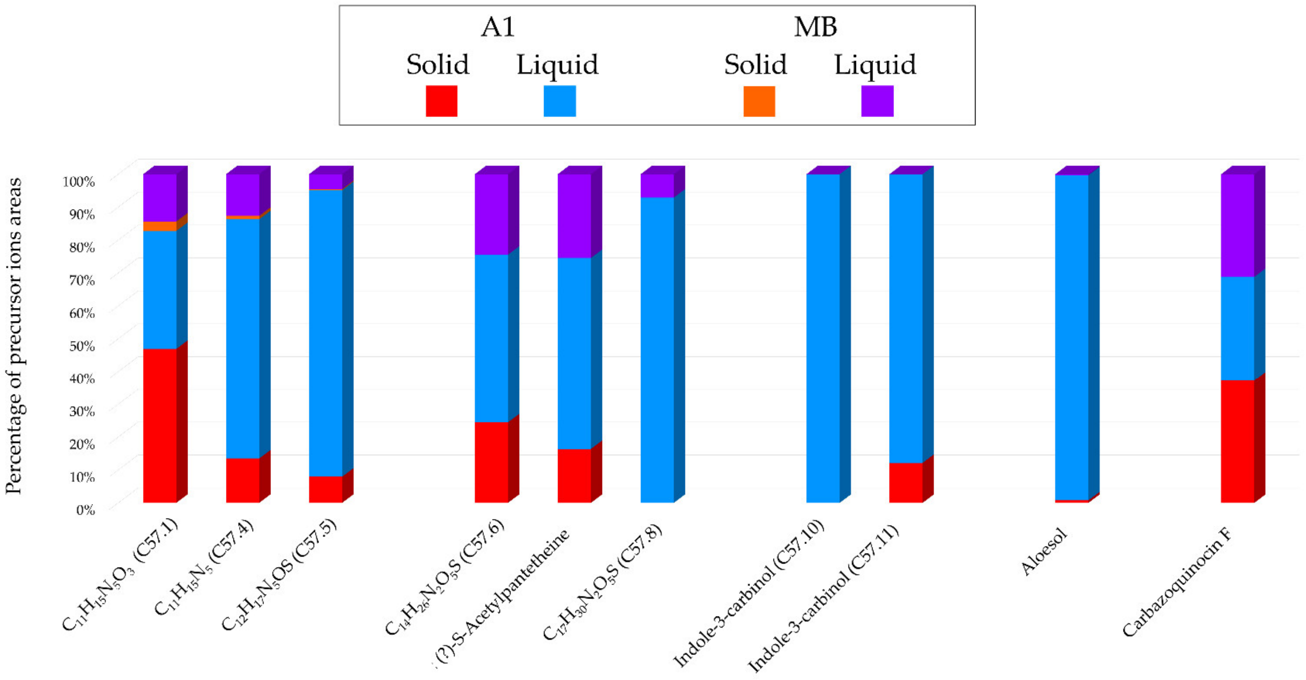

Figure S10. *Micromonospora* sp. SH-57 : Main annotations in the IIMN designed from the extracts of A1 or MB solid and liquid cultures at 14 days. The histograms present the cumulative proportions relative to the precursor ions areas intensities attributed to each culture condition.

Table S5. Summary table of annotations from the Ion Identity Molecular Network of the different extracts of *Micromonospora* sp. SH-57.

**Compound ID**	**RT**	**m/z [+Adduct]**	**Compound name or InChIKey (1,2,3)**	**Precursor ions areas observed in MzMine (with the maximum in bold) according to the culture conditions**								
				**Medium**	**A1**						**MB**	
				**Support**	**Solid**			**Liquid**			**Solid**	**Liquid**
				**Days**	**7**	**14**	**21**	**7**	**14**	**21**	**14**	
C57.1	1.08	266.1245 $[M+H]^+$	2-Deoxy-*N*6-methyladenosine (1)		5,0E+02	9,8E+02	7,5E+02	1,7E+03	7,5E+02	**3,8E+03**	6,0E+01	3,0E+02
C57.2	3.45	234.1351 $[M+H]^+$	SXIDRQQQIPLCTJ (1)		2,0E+02	2,0E+02	2,0E+02	2,0E+03	2,0E+03	**6,8E+03**	-	-
C57.3	8.48	264.1277 $[M+H]^+$	UHYRJPGYRFMFLT (1)		9,9E+02	4,1E+02	1,5E+03	8,9E+03	9,1E+03	**1,4E+04**	-	-
C57.4	6.08	218.1400 $[M+H]^+$	9-cyclopentyl-*N*-methylpurin-6-amine (1)		5,5E+03	3,7E+03	4,1E+03	2,1E+04	2,0E+04	**4,5E+04**	2,8E+02	3,4E+03
C57.5	7.12	280.1225 $[M+H]^+$	INPAYTORGXXLMB (1)		3,7E+03	2,4E+03	6,4E+03	3,5E+04	2,7E+04	**5,2E+04**	1,0E+02	1,4E+03
C57.6	6.19	335.163 $[M+H]^+$	JDNYVZBVEBRRCT (1)		2,0E+02	3,0E+02	3,0E+02	5,0E+02	6,3E+02	**2,4E+03**	-	3,0E+02
C57.7	5.38	343.1292 $[M+Na]^+$	(?)-*S*-Acetylpantetheine (1)		4,0E+02	1,0E+03	1,5E+03	6,2E+03	3,7E+03	**7,0E+03**	-	1,6E+03
C57.8	7.31	375.1949 $[M+H]^+$	ZCNIMMSEOJFZKZ (1)		-	-	-	2,0E+02	1,3E+03	**8,2E+03**	-	1,0E+02
C57.9	7.32	397.1772 $[M+H]^+$	IXKOTSUCYPEFPP (1)		-	1,0E+02	2,0E+02	8,0E+02	2,0E+03	**5,1E+03**	-	2,0E+02
C57.10	8.53	130.0652 $[M+H-H_2O]^+$	Indole-3-carbinol (1,2)		-	-	-	6,0E+02	6,3E+02	**1,3E+03**	-	-
C57.11	8.40	130.0653 $[M+H-H_2O]^+$			1,0E+02	1,0E+02	1,0E+02	5,0E+02	7,2E+02	**1,4E+03**	-	-
C57.12	6.72	233.0812 $[M+H]^+$	Aloesone (1)		-	5,0E+01	1,0E+02	**6,9E+03**	3,1E+03	1,7E+03	-	-
C57.13	6.52	235.0967 $[M+H]^+$	Aloesol (1)		5,0E+01	5,0E+01	5,0E+01	2,5E+03	6,2E+03	**1,7E+04**	-	2,0E+01
C57.14	9.70	310.1803 $[M+H]^+$	Carbazoquinocin C (1,3)		-	-	9,6E+02	8,0E+02	8,0E+02	**6,2E+03**	-	-
C57.15	10.00	324.1958 $[M+H]^+$	Carbazoquinocin E (1,3)		-	-	-	-	-	**1,5E+03**	-	-
C57.16	10.30	338.2111 $[M+H]^+$	Carbazoquinocin F (1,3)		5,0E+02	7,2E+02	4,5E+03	5,9E+02	6,1E+02	**1,5E+04**	-	6,0E+02
C57.17	10.60	366.2423 $[M+H]^+$	12-Carbazol-9-yldodecanoic acid (1)		-	-	-	-	5,0E+02	**7,7E+02**	-	-

Data from [1] SIRIUS, [2] GNPS or [3] ISDB timaR bioinformatics tools.

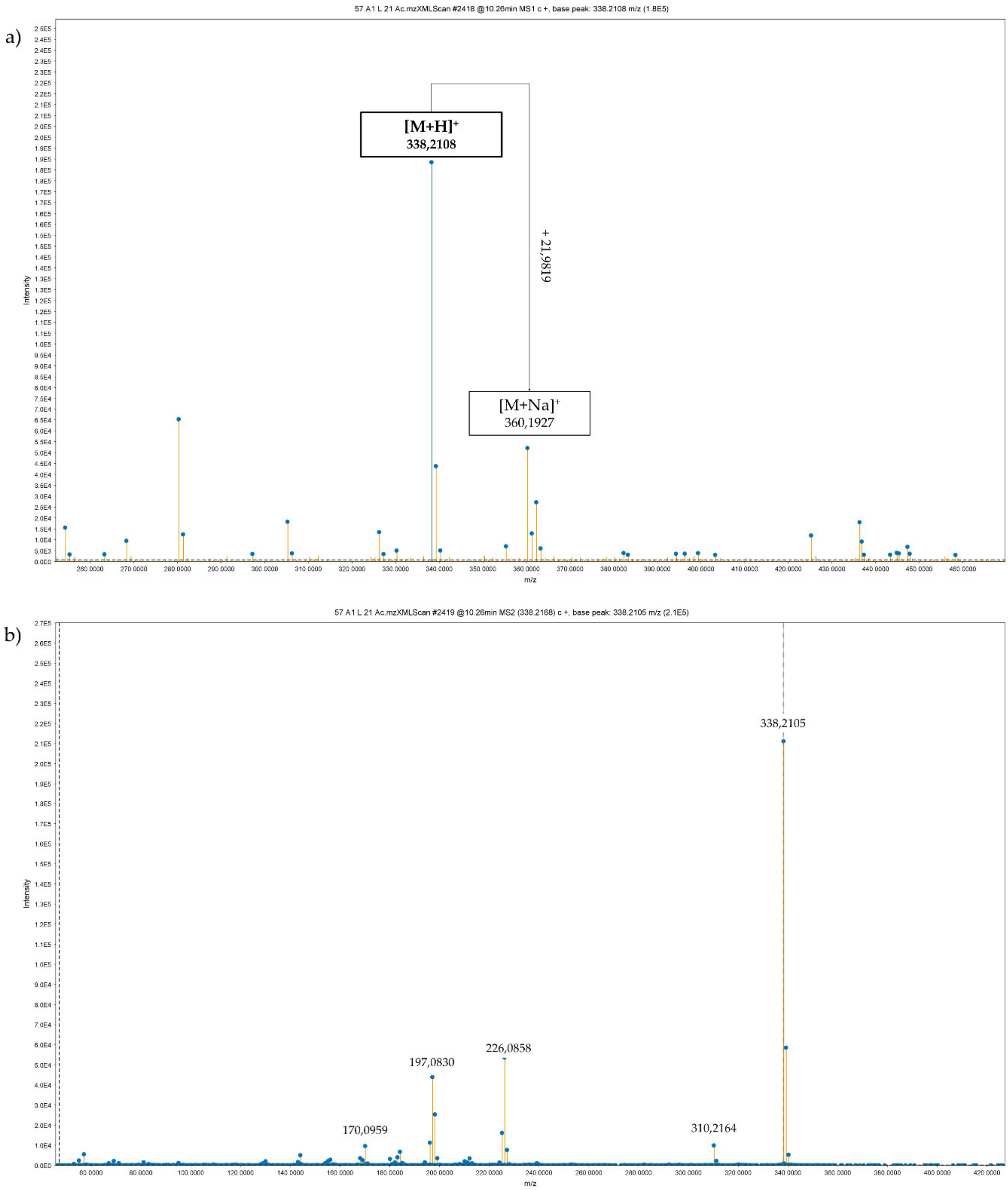

Figure S11. Spectral data of carbazoquinocin F
(C 57.16, *m*/z 338.2113, [M+H]$^+$, $C_{22}H_{27}NO_2$) : (a) MS1 and (b) MS2.

Table S6. Detailed of observed peaks in the HPLC-CAD chromatographic profiles of microbials extracts from *Salinispora arenicola* SH-78.

**Culture conditions**					
**Medium**		A1			
**Support**		Solide			Liquid
**Days**		7	14	21	14
**Code extract**		78 A1 S 7 Ac	78 A1 S 14 Ac	78 A1 S 21 Ac	78 A1 L 14 Ac

**Peaks code**	**Retention Time (min)**	**Height of peaks (pA)**			
		**78 A1 S 7 Ac**	**78 A1 S 14 Ac**	**78 A1 S 21 Ac**	**78 A1 L 14 Ac**
P 78.1	13.4	12	16	12	10
P 78.2	20.3	5	10	5	9
P 78.3	20.8	7	11	5	11
P 78.4	24.8	10	10	6	15
P 78.5	26.8	10	17	7	12
P 78.6	28.3	-	-	-	38
P 78.7	29.5	8	12	6	15
P 78.8	34.5	10	15	5	96

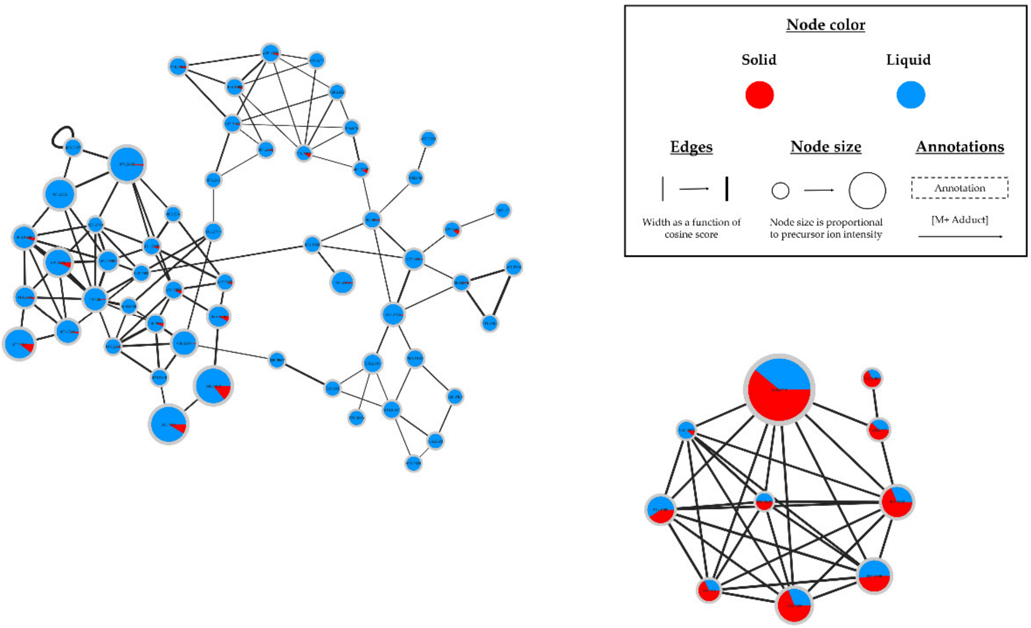

Figure S12. Zoom-in on two largest cluster of *Salinispora arenicola* SH-78 is not clearly annotated.

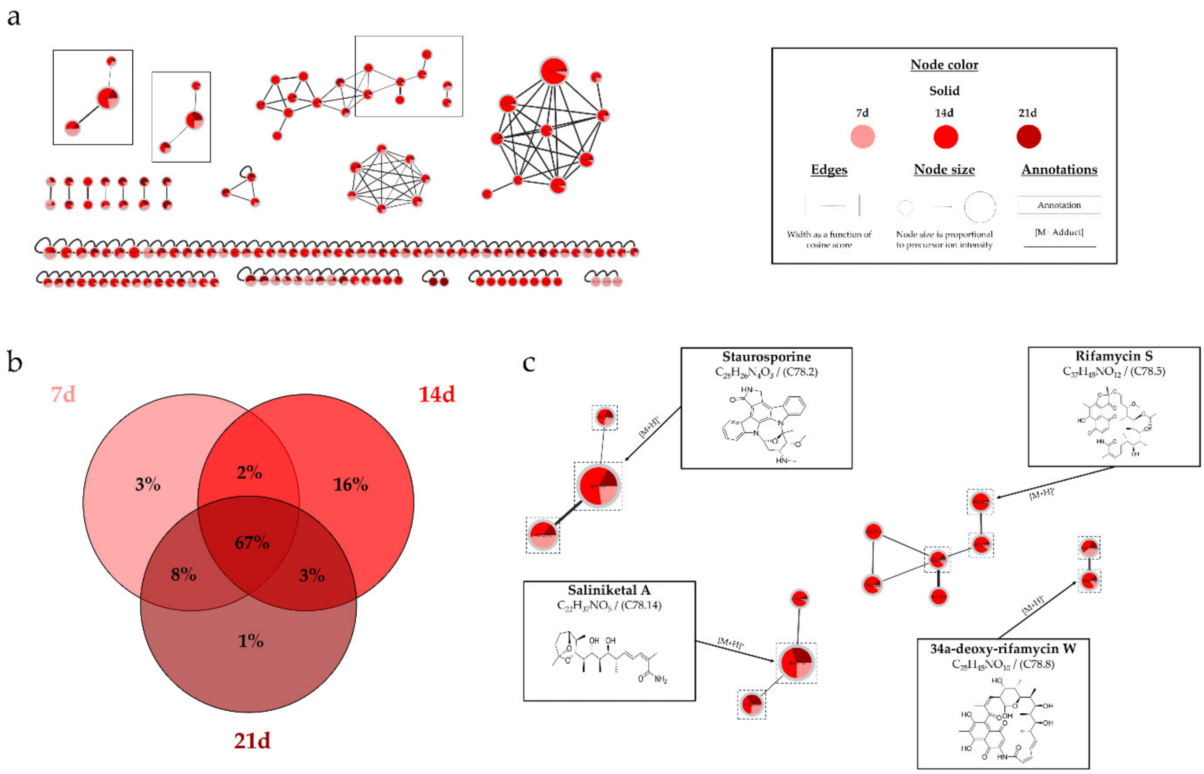

Figure S13. *Salinispora arenicola* SH-78 : (a) Ion Identity Molecular Network (IIMN) from the extracts of A1 solid (red gradient) cultures at 7, 14 and 21 days. (b) Percentages of nodes as a function of duration. (c) Zoom on 3 annotated clusters of interest.

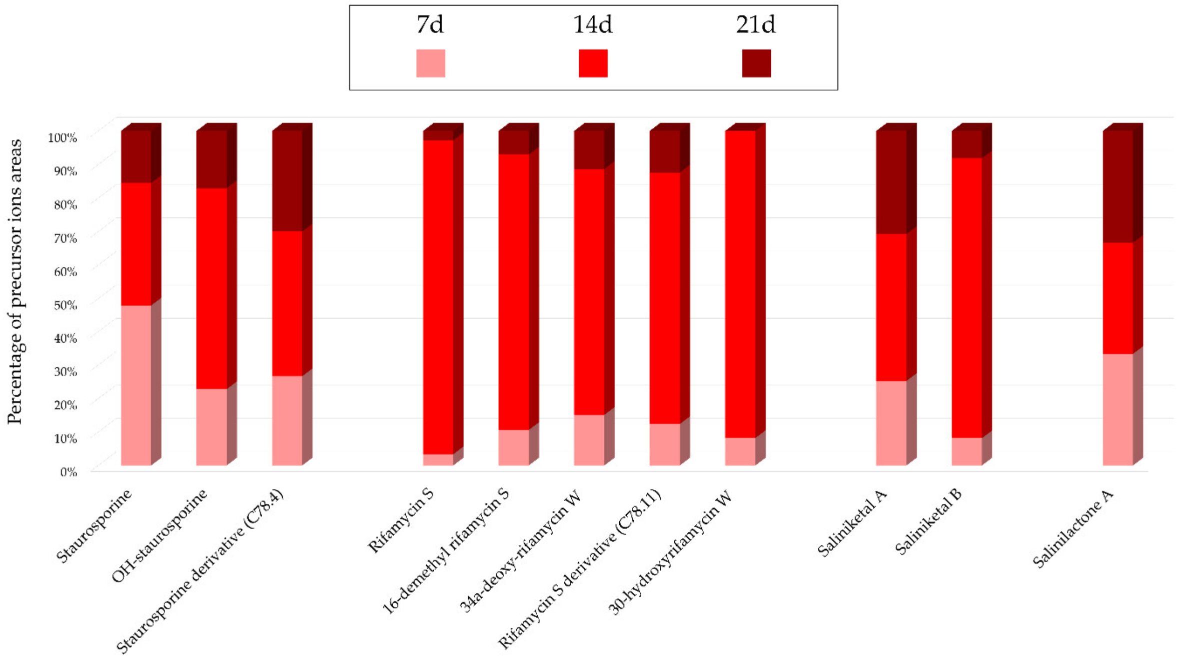

Figure S14. *Salinispora arenicola* SH-78 : Main annotations in the IIMN designed from the extracts of A1 solid cultures at 7, 14 and 21 days. The histograms present the cumulative proportions relative to the precursor ions areas intensities attributed to each culture condition.

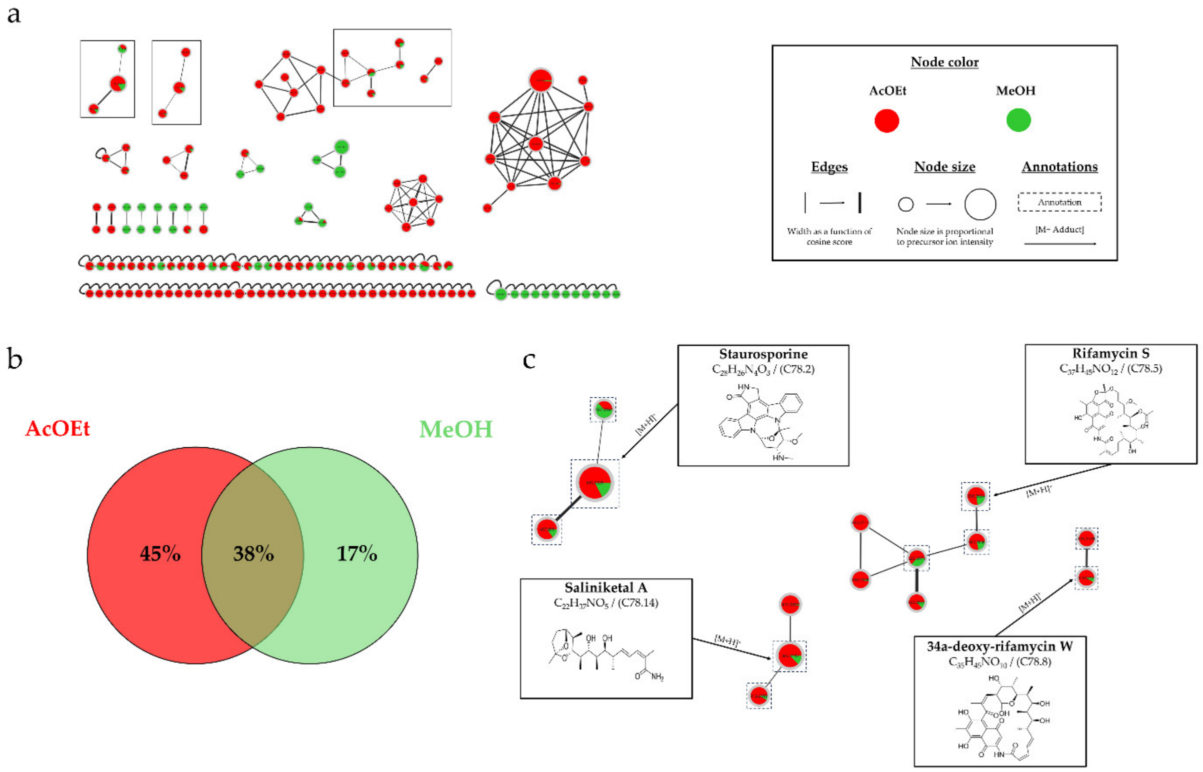

Figure S15. *Salinispora arenicola* SH-78 : (a) Ion Identity Molecular Network (IIMN) from AcOEt (red) and MeOH extracts of the A1 solid medium culture at 14 days. (b) Percentages of nodes as a function of extractions' solvent. (c) Zoom on 3 annotated clusters of interest.

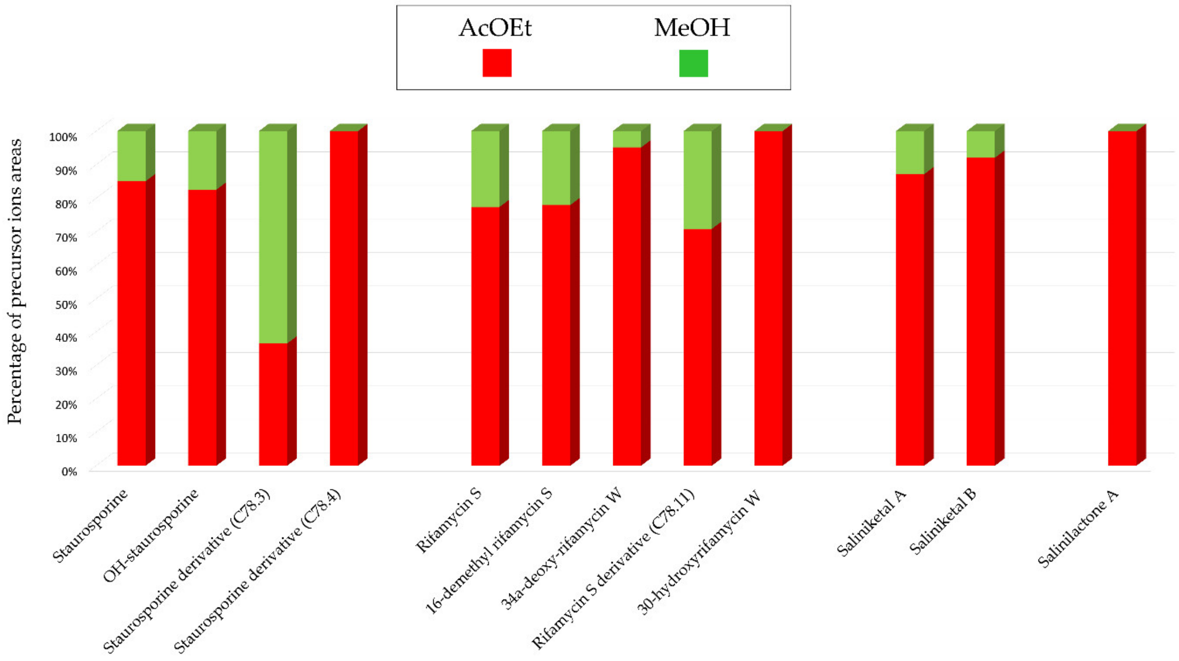

Figure S16. *Salinispora arenicola* SH-78 : Main annotations in the IIMN designed from AcOEt and MeOH extracts from the A1 solid medium culture at 14 days. The histograms present the cumulative proportions relative to the precursor ions areas intensities attributed to each extraction condition.

Table S7. Summary table of annotations from the Ion Identity Molecular Network of the different extracts of *Salinispora arenicola* SH-78.

Compound ID	RT	m/z [+Adduct]	Compound name or InChIKey [1,2,3]	Precursor ions areas observed in MzMine (with the maximum in bold) according to the culture conditions					
				Solvant	AcOET				MeOH
				Support	Solid			Liquid	Solid
				Days	7	14	21	14	14
C78.1	7,20	483,2028 [M+H]+	OH staurosporine [1,2,3]		2,4E+04	**6,2E+04**	1,8E+04	4,5E+03	1,3E+04
C78.2	7,33	467,2085 [M+H]+	Staurosporine [1,2,3]		**2,4E+04**	1,8E+04	7,6E+03	3,0E+03	3,2E+03
C78.3	7,30	497,2192 [M+H]+	4'-*N*-methyl-5'-hydroxy-staurosporine [1]		1,3E+03	2,5E+03	9,6E+02	9,9E+02	**4,3E+03**
C78.4	7,53	497,1825 [M+H]+	4'-demethyl-Af-formyl-7V-hydroxy-staurosporine [1]		4,0E+02	**6,5E+02**	4,5E+02	-	-
C78.5	9,70	696,3022 [M+H]+	Rifamycin S [1,2,3]		3,0E+02	8,4E+03	2,5E+02	**1,6E+04**	2,5E+03
C78.6	9,24	682,2866 [M+H]+	16-demethyl rifamycin S [3]		4,5E+02	**3,5E+03**	3,0E+02	3,2E+03	9,9E+02
C78.7	8,40	640,3113 [M+H]+	34a-deoxy-rifamycin W [1]		-	-	-	**2,0E+03**	-
C78.8	9,75	640,3119 [M+H]+			4,0E+02	1,9E+03	3,0E+02	**7,5E+02**	1,0E+02
C78.9	8,30	624,3190 [M+H]+	Proansamycin B [1,3]		1,0E+03	**1,0E+03**	5,0E+02	3,0E+02	-
C78.10	9,39	624,3164 [M+H]+			3,0E+02	1,4E+03	1,0E+02	**2,5E+03**	-
C78.11	8,73	622,2658 [M-H$_2$O+H]+	Demethyl-desacetyl-rifamycin S [1]		2,0E+02	1,2E+03	2,0E+02	**1,2E+04**	5,0E+02
C78.12	9,57	712,2971 [M+H]+	20-hydroxyrifamycin S [1,3]		-	-	-	**1,9E+03**	-
C78.13	7,61	672,3036 [M+H]+	30-hydroxyrifamycin W [1]		5,0E+01	5,6E+02	-	**7,4E+02**	-
C78.14	7,84	396,2745 [M+H]+	Saliniketal A [1,3]		1,8E+04	3,1E+04	2,2E+04	**3,3E+04**	4,6E+03
C78.15	6,90	412,2693 [M+H]+	Saliniketal B [1,3]		5,4E+03	**7,7E+03**	6,9E+03	6,6E+03	7,0E+02

Data from [1] SIRIUS, [2] GNPS or [3] ISDB timaR bioinformatics tools.

Table S7. *Continued*

Compound ID	RT	m/z [+Adduct]	Compound name or InChIKey [1,2,3]	Solvant	AcOET				MeOH
				Support	Solid			Liquid	Solid
				Days	**7**	**14**	**21**	**14**	**14**
C78.16	6,47	155,0703 [M+H]+	Salinilactone D [3]		8,0E+02	3,5E+02	6,0E+02	**4,0E+04**	-
C78.17	6,88	169,0858 [M+H]+	Salinilactone E [3]		-	6,0E+02	-	**3,4E+04**	-
C78.18	7,28	169,086 [M+H]+			9,0E+03	7,6E+03	7,3E+03	**1,1E+05**	2,0E+02
C78.19	7,67	183,1016 [M+H]+	Salinilactone A [3]		3,0E+02	3,0E+02	3,0E+02	**7,0E+03**	-
C78.20	7,79	183,1016 [M+H]+			4,0E+02	4,0E+02	4,0E+02	**8,6E+03**	-
C78.21	7,95	183,1017 [M+H]+			4,0E+02	4,0E+02	4,0E+02	**1,0E+04**	-
C78.22	8,44	197,1174 [M+H]+	Salinilactone C [3]		3,0E+03	3,7E+03	2,5E+03	**1,4E+04**	-
C78.23	8,84	211,1332 [M+H]+	Salinilactone H [3]		1,0E+03	2,0E+03	8,0E+02	**1,5E+04**	-

Header spanning the culture-condition columns: **Precursor ions areas observed in MzMine (with the maximum in bold) according to the culture conditions**

Data from [1] SIRIUS, [2] GNPS or [3] ISDB timaR bioinformatics tools.

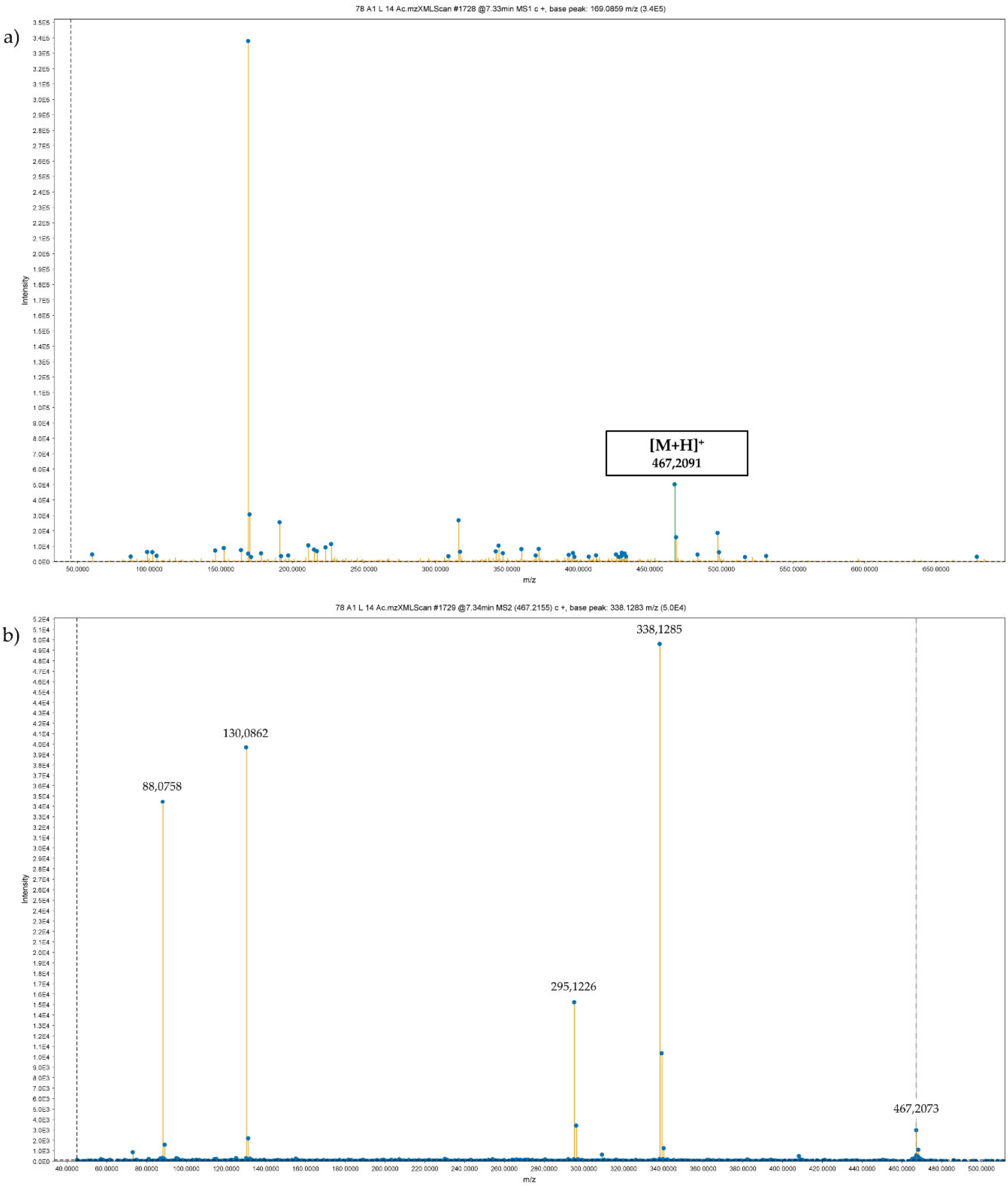

Figure S17. Spectral data of staurosporine
(C78.2, *m/z* 467.2085 [M+H]$^+$, $C_{28}H_{26}N_4O_3$) : (a) MS1 and (b) MS2.

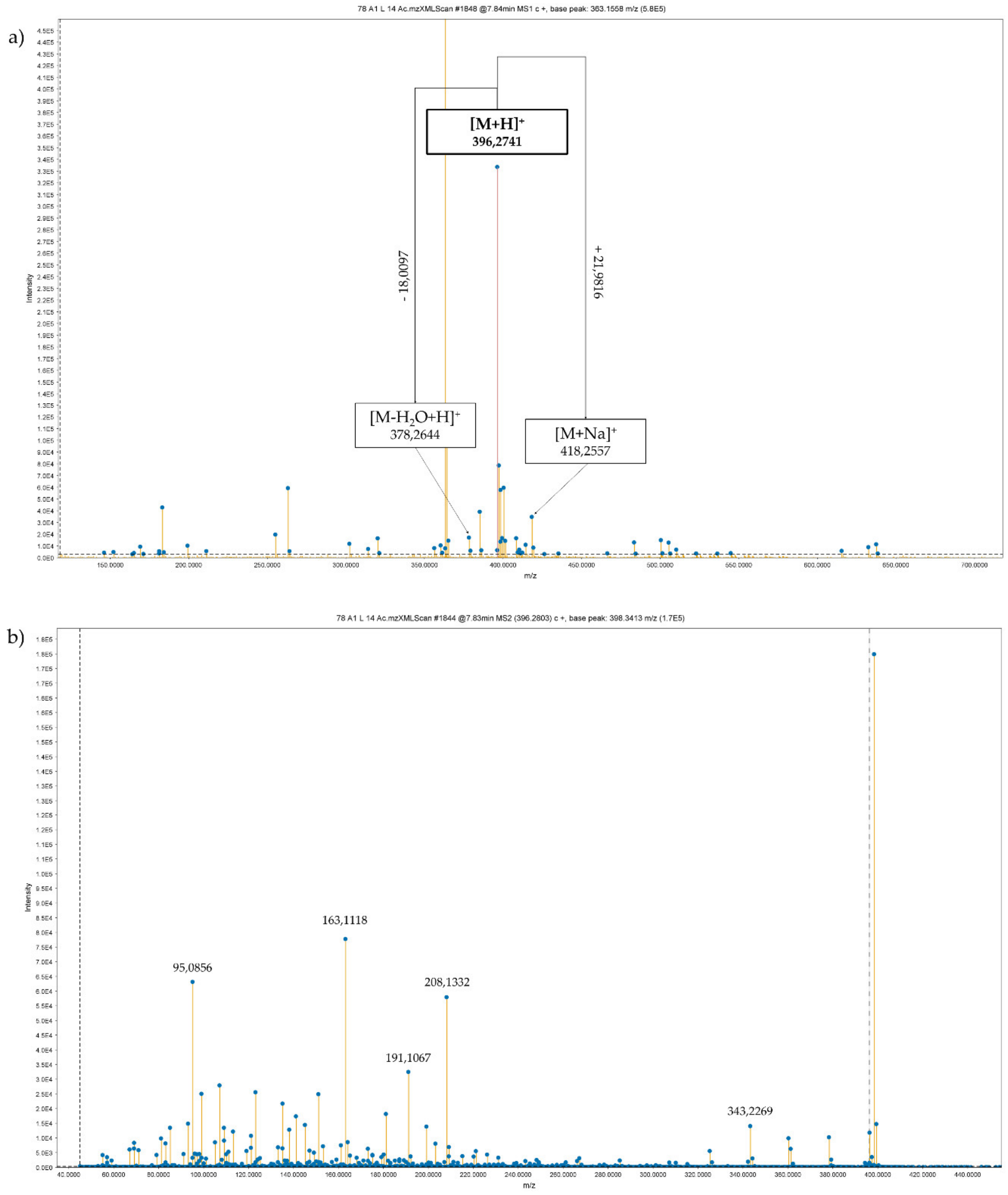

Figure S18. Spectral data of saliniketal A (C78.14, m/z 396.2745 [M+H]+, $C_{22}H_{37}NO_5$) : (a) MS1 and (b) MS2.

Table S8. Batch mode use for data processing with MzMine 3 software.

1) Mass detection

Scan MS1 centroid ; noise level : 3^E3

Scan MS2 centroid ; noise level : 0^E0

2) ADAP Chromatogram Builder

Min group size in # of scans : 3

Group intensity threshold : 3^E3

Min highest intensity : 5^E3

Scan to scan accuracy : 0.0050 *m/z* or 20 ppm

3) Local minimum feature resolver

MS/MS scan pairing: retention time (RT) tolerance : 0.2 min

MS1 to MS2 precursor tolerance : 0.0050 *m/z* or 20 ppm

Chromatographic threshold : 85%

Minimum search range RT : 0.080 min

Minimum relative height : 0%

Minimum absolute height : 2.503

Min ratio of peak top/edge : 1.7

Peak duration range: 0-2 min

Min # of data points : 3

4) 13C isotope filter

m/z tolerance : 0.005 *m/z* or 20 ppm

RT tolerance : 0.08 min

Maximum charge : 2

Representative isotope most intense

5) Join aligner

m/z tolerance : 0.005 *m/z* or 20 ppm

Weight for *m/z* : 3

RT tolerance : 0.08 min

Weight for RT : 1

6) Feature list blank subtraction

Minimum # of detection in blanks : 1

7) metaCorrelate

RT : 0.05 min

Min height : 3^E3

Intensity correlation threshold : 3^E3

Correlation grouping

Feature height correlation

8) Ion identity molecular networking

m/z tolerance 0.005 *m/z* or 20 ppm

Min height 3^E3

9) Export feature list GNPS

Feature intensity : peak area

CSV export simple

Table S9. Parameters use for molecular network with GNPS.

1) Basic options

Precursor Ion Mass Tolerance : 0.02 Da

Fragment Ion Mass Tolerance : 0.02 Da

2) Advanced network options

Min paris cos : 0.70

Network TopK : 7

Minimum Matched Fragment Ions : 12

3) Advanced Library Search Options

Library Search Min Matched Peaks : 6

Score Threshold : 0.7
